# Role of *SFP1* in the Regulation of *Candida albicans* Biofilm Formation

**DOI:** 10.1371/journal.pone.0129903

**Published:** 2015-06-18

**Authors:** Hsueh-Fen Chen, Chung-Yu Lan

**Affiliations:** 1 Institute of Molecular and Cellular Biology, National Tsing Hua University, Hsinchu, 30013, Taiwan, R.O.C; 2 Department of Life Science, National Tsing Hua University, Hsinchu, 30013, Taiwan, R.O.C; Institute of Microbiology, SWITZERLAND

## Abstract

*Candida albicans* is a major human fungal pathogen. One of the important features of *C*. *albicans* pathogenicity is the ability to form biofilms on mucosal surfaces and indwelling medical devices. Biofilm formation involves complex processes in *C*. *albicans*, including cell adhesion, filamentous growth, extracellular matrix secretion and cell dispersion. In this work, we characterized the role of the transcription factor Sfp1, particularly with respect to its function in the regulation of biofilm formation. The deletion of the *SFP1* gene enhanced cell adhesion and biofilm formation in comparison to the wild-type strain. Interestingly, the *sfp1*-deleted mutant also exhibited an increase in the expression of the *ALS1*, *ALS3* and *HWP1* genes, which encode adhesin proteins. In addition, Sfp1 was demonstrated to function downstream of the Rhb1-TOR signaling pathway. Bcr1 and Efg1 are transcription factors that are critical for controlling biofilm formation, and Efg1 is also required for hyphal growth. Deleting either the *BCR1* or *EFG1* gene in the *sfp1*-null background led to reduced adhesin gene expression. As a result, the *bcr1*/*sfp1* or *efg1*/*sfp1* double deletion mutants exhibited dramatically reduced biofilm formation. The results indicated that Sfp1 negatively regulates the *ALS1*, *ALS3* and *HWP1* adhesin genes and that the repression of these genes is mediated by the inhibition of Bcr1 and Efg1.

## Introduction

*Candida albicans* is a part of the normal microbial flora and typically inhabits the skin, the mucosal surfaces of the oral cavity, and the gastrointestinal and genitourinary tracts. However, this organism is also an opportunistic pathogen that can cause invasive and life-threatening infections, particularly in immunocompromised patients [1]. A critical feature that is closely related to the ability of *C*. *albicans* to cause infections is its ability to form surface-associated microbial communities called biofilms [2–4]. *C*. *albicans* can form biofilms on biotic or inert surfaces, such as the mucosal epithelia and a variety of indwelling medical devices. The increased use of implanted medical devices has enhanced the risk of *C*. *albicans* biofilm-related infections; thus, *C*. *albicans* has emerged as a major causative agent of nosocomial infections [2,4–6]. For example, biofilm formation on intravascular catheters is a key step in the development of hematogenously disseminated candidiasis [7]. Moreover, *C*. *albicans* is ranked third among the leading causes of intravascular catheter-related infections with high levels of mortality [5,8–11].

Complex processes for biofilm formation in *C*. *albicans* have been proposed. Biofilm formation begins with the adhesion of yeast cells to a supporting surface, which is followed by cell proliferation that forms a basal layer along the surface [12]. Filamentous growth is then induced, and the biofilm is enclosed by secreted extracellular matrix materials, forming a complex three-dimensional architecture [11–13]. Finally, cells disperse from the mature biofilm into the surroundings. Biofilms protect *C*. *albicans* from antifungals and may also help cells escape from the host immune system [14–17]. The antifungal resistance of *C*. *albicans* remains a great threat in clinical settings. Therefore, to understand *C*. *albicans* pathogenesis and develop novel antifungal strategies, it is necessary to elucidate different aspects of biofilm formation.

In addition, *C*. *albicans* biofilm formation involves complex signaling and gene regulation networks [11,18]. The identification and study of transcription factors related to biofilm formation should provide important insights into the molecular mechanisms that control biofilm formation. For example, the deletion of the *BCR1* gene or the *MSS11* gene, which both encode transcription factors, affects biofilm formation in *C*. *albicans* [19,20]. Moreover, using a computational approach, we constructed the gene regulatory networks of biofilm and planktonic cells [21]. By comparing the network structure and performing statistical analysis, we revealed the differences between the networks and identified several potential transcription factors related to biofilm formation [21]. Among those candidates, orf19.5953 exhibited the most statistical significance and was considered to be a transcription factor closely related to biofilm formation. In this study, we characterized the function of *C*. *albicans* orf19.5953. We demonstrated that *C*. *albicans* orf19.5953 is a functional homolog of *Saccharomyces cerevisiae* Sfp1. Moreover, the deletion of *C*. *albicans SFP1* promotes biofilm formation in comparison to the wild-type (WT) strain. The *SFP1*-deleted mutant also exhibited higher expression of the *ALS1*, *ALS3* and *HWP1* genes, which encode cell wall adhesion proteins [22–24]. Finally, Sfp1 functions downstream of the Rhb1-Tor1 signaling pathway and may coordinate with the transcriptional factors Bcr1 and Efg1. Our findings highlight a previously unknown mechanism for signaling and transcription regulation during *C*. *albicans* biofilm formation.

## Materials and Methods

### Yeast strains and growth conditions

All of the *C*. *albicans* strains used in this study are listed in Table A in S1 File. The cells were routinely grown in YPD medium (2% glucose, 1% yeast extract and 2% peptone). Plates were prepared with 1.5% agar. For the assay of cell adhesion and biofilm formation, synthetic complete (SC) medium (0.67% yeast nitrogen base [YNB] with ammonium sulfate, 2.0% glucose, and 0.079% complete supplement mixture) was used.

### DNA manipulation and strain construction

#### (1) Deletion and reintegration of the *C. albicans SFP1, EFG1* and *BCR1* genes

All deletion strains were generated from SC5314 using the *SAT1*-flipper method [25]. The primers used are listed in Table B in S1 File. The 5’ flanking region of *SFP1* was amplified from the SC5314 genome using the primer pair CaSFP1uF-KpnI and CaSFP1uR-XhoI. The 3’ flanking region of *SFP1* was amplified from the SC5314 genome using the primer pair CaSFP1dF-SacII and CaSFP1dR-SacI. The resulting 5’ and 3’ flanking regions of *SFP1* were independently cloned into the pSFS2A vector [25] to generate pSFS2AdSFP1. The DNA fragment carrying the 5’ and 3’ flanking regions of *SFP1* and the *SAT1*-flipper cassette was excised from pSFS2AdSFP1 via *Kpn*I/*Sac*I digestion. The linear DNA was purified and transformed into *C*. *albicans* cells for integration into the chromosome between the 5’ and 3’ flanking sequences of *SFP1* via homologous recombination. The transformants were selected for nourseothricin resistance and verified by PCR. To remove the integrated *SAT1*-flipper cassette from the *SFP1* locus, the cells were grown in YPM medium (2% maltose, 1% yeast extract and 2% peptone) to induce the *MAL2* promoter-regulated recombinase for *SAT1*-FLIP excision. The heterozygous *sfp1*-deleted mutants (*sfp1*Δ/*SFP1*) were used for a second round of deletion cassette integration and excision to knock out the second allele of *SFP1*. Two independently generated heterozygous and homozygous *sfp1*-deleted mutants (*sfp1*Δ/*sfp1*Δ) were used for further study.

To construct the *SFP1*-reintegrated strain, the DNA fragment composed of the *SFP1* promoter along with the full-length *SFP1* coding sequence was amplified from the SC5314 genome using the primer pair CaSFP1uF-KpnI and CaSFP1-2R-XhoI. This fragment was cloned into pSFS2AdSFP1 upstream of the *SAT1*-flipper cassette to replace the original *Kpn*I-*Xho*I fragment, generating pSFP1R. The DNA fragment carrying the full-length *SFP1* gene, the *SAT1*-flipper cassette and the 5’ and 3’ flanking regions of *SFP1* was excised from pSFP1R via *Kpn*I/*Sac*I digestion, purified and transformed into the homozygous *SFP1*-deleted strains. Nourseothricin selection and removal of the *SAT1*-cassette were performed as described above. The strains carrying the integration in the first allele of *SFP1* were used to integrate *SFP1* into the second allele. Two independent *SFP1*-reintegrated strains were used in different experiments.

Similar procedures were used to delete and reintegrate *EFG1* and *BCR1* in the homozygous *sfp1*-deleted background, generating the *efg1*Δ/*efg1*Δ*/sfp1*Δ/*sfp1*Δ and *bcr1*Δ/*bcr1*Δ*/sfp1*Δ/*sfp1*Δ double mutant strains. The primer pair EFG1-UR-F-KpnI and EFG1-UR-R-XhoI was used for the 5’ flanking region of the *EFG1* gene, and the primer pair EFG1-DR-F-SacII and EFG1-DR-R-SacI was used for the 3’ flanking region of the *EFG1* gene. In addition, the primer pair BCR1 UR F1-KpnI and BCR1 UR R2-XhoI was used for the 5’ flanking region of *BCR1*, and the primer pair BCR1 DR F1-SacII and BCR1 DR R2-SacI was used for the 3’ flanking region of *BCR1*.

#### (2) Overexpression of the *C. albicans SFP1* gene

To construct a strain capable of tetracycline-induced *SFP1* overexpression, the *GFP* gene was excised from the pNIM1 plasmid via *Bgl*II/*Sal*I digestion [26] and replaced with the full-length *SFP1* sequence, generating pNIM1-SFP1. A DNA fragment containing the tetracycline-inducible promoter (*P*tet)-*SFP1*-*SAT1* cassette was obtained from pNIM1-SFP1 via *Sac*II/*Kpn*I digestion. This linear DNA fragment was purified and transformed into the SC5314 genome for integration into one allele of the *ADH1* locus.

### One-hybrid assay

To generate the strains used in the one-hybrid assay, the *SFP1* gene was PCR-amplified from the SC5314 genome using the primer pair SFP1-one-hybrid-1 and SFP1-one-hybrid-2 (Table B in S1 File). The PCR product was cloned into the pCIp-lexA-F1 plasmid [27,28] between the *Mlu*I and *Sph*I restriction enzyme sites to generate pCIp-LexA-F-SFP1. The DNA fragment containing the *lexA*-*SFP1* fusion was obtained from pCIp-LexA-F-SFP1 via *Stu*I digestion, purified and introduced into the *RPS1* locus of the COP1 and CCR1 strains [28] to yield COP-LexASFP1 and CCR-LexASFP1, respectively (Table A in S1 File). The COP1-derived strains use a LexA operator and an *ADH1* basal promoter to drive the *lacZ* reporter, whereas the CCR1-derived strains lack the LexA operator.

The expression level of β-galactosidase was determined using an X-Gal (5-bromo-4-chloro-3-indolyl-β-D-galactopyranoside) overlay assay and a liquid β-galactosidase assay, as described previously [28], with some modifications. For the overlay assays, colonies were formed on agar plates during an overnight incubation and lysed with chloroform for 5 min. We decanted the chloroform solution and air-dried the plates for 10 min. The plates were overlaid with X-Gal—agarose (0.25 or 0.5 mg/ml of X-Gal, 0.1 M sodium phosphate buffer [pH 7.0] and 1% agarose). After the gel solidified, the plates were incubated overnight at 37°C until the blue color developed. For the liquid β-galactosidase assays, the cells were grown overnight in YPD at 30°C, subcultured into fresh YPD and grown to mid-log phase (~5 h). The cell pellets were collected, washed with sterile double-distilled water (ddH_2_O), and stored at -80°C until use. The cells were resuspended in 300 μl of Z buffer (60 mM Na_2_HPO_4_, 40 mM NaH_2_PO_4_, 10 mM KCl, 1 mM MgSO_4_·7H_2_O, and 4 μl/ml of 98% mercaptoethanol, pH 7.0). The cell suspension was divided equally into three tubes, and 900 μl of Z buffer were added to each tube. The cells were lysed by adding 15 μl of 0.1% SDS and 30 μl of chloroform and vortexing the samples for 15 sec. Then, 0.2 ml of 4 mg/ml o-nitrophenyl-β-D-galactopyranoside (ONPG) in potassium phosphate buffer (pH 7.0) was added and the mixture was incubated at 37°C for 30 min. When the mixture turned yellow, the reaction was stopped via the addition of 400 μl of 1 M Na_2_CO_3_. The cell debris was removed by centrifugation, and the absorbance of the supernatants was measured at 420 and 550 nm. The β-galactosidase activity was calculated as follows: 1 Miller Unit = 1000 × [(OD_420_)–(1.75 × OD_550_)] / [(*t*) × (*v*) × (OD_600_)], where OD_420_ is the absorbance derived from ONPG, *t* is the duration of the reaction (in minutes), *v* is the volume of the supernatant used in the assay (in milliliters), OD_600_ is the cell density at the beginning of the reaction, and OD_550_ is the light scattering from cell debris. The assays were performed in triplicate, with at least three independent experiments for each tested strain.

### RNA preparation and reverse transcription (RT) real-time quantitative PCR (qPCR)

The biofilm cells were scraped from the microplates and washed with ddH_2_O. The cell pellets were collected via centrifugation and stored at -80°C until use. Total RNA extraction and cDNA synthesis were performed as described previously [27]. The *PMA1* transcripts were used as an internal control for the RNA input [29].

Real-time qPCR was performed using the StepOne Plus real-time PCR system (Applied Biosystems, Framingham, MA, USA). The primers used are listed in Table B in S1 File. Briefly, each 20 μl reaction mixture contained 80 ng of cDNA, 300 nM of each forward and reverse primer, and 10 μl of the Power SYBR green PCR master mixture (Applied Biosystems). The reactions were performed with 1 cycle at 95°C for 10 min, followed by 40 repeated cycles at 95°C for 15 sec and 60°C for 1 min. The *PMA1* transcripts were used as an endogenous control for qPCR [29]. All experiments were repeated independently at least two times, and the average C_T_ values were determined. The relative fold change in the expression of each gene was calculated using the 2^–ΔΔCT^ method [30].

### Cell susceptibility to rapamycin and hygromycin B

Cells from overnight cultures were harvested via centrifugation, washed and diluted to 3 × 10^7^ cells per ml in sterile ddH_2_O. Five microliters of 10-fold serial dilutions were spotted onto YPD agar plates containing 25 ng/ml of rapamycin (catalog no. 553210; Merck KGaK, Darmstadt, Germany). The rapamycin stock solution (100 μg/ml) was prepared in methanol. In addition, cells were grown on YPD plates that contained the same volume of methanol (without rapamycin) and used as controls. For the assay of cell susceptibility to hygromycin B, YPD agar plates with or without 400 μg/ml of hygromycin B (catalog no. H7772-250MG; SIGMA) were used. Cell viability was recorded after incubation at 30°C for 5 days. The cell susceptibility assay was performed independently 3 times.

### Measurement of cell adhesion and biofilm formation

The WT, *SFP1*-deleted and *SFP1*-reintegrated strains of *C*. *albicans* were grown overnight in YPD broth at 30°C. The overnight culture was subcultured into fresh SC medium and grown for 5 h. For the cell adhesion assay, 10^7^ cells were suspended in 0.25 ml of SC medium/per well of a 24-well flat polystyrene microplate (Cat number 4430300, Orange Scientific, Braine-l’Alleud, Belgium) and incubated for 1 h at 37°C with 5% CO_2_. The plate was washed twice with sterile phosphate-buffered saline (PBS). Adhered cells were evaluated by assessing the reduction of 2,3-bis (2-methoxy-4-nitro-5-sulfophenyl)-2H-tetrazolium-5-carboxanilide (XTT) and by measuring the optical density at 600 nm (OD_600_). For the XTT reduction assay, XTT (1 mg/ml) and menadione (14.28 μM) were added to each well and the samples were incubated at 37°C for 20 minutes. The reaction was measured at 490 nm using a microplate reader [31]. For OD_600_ measurement, adherent cells were scraped from each well and resuspended in PBS and the OD_600_ was measured [32].

For the assay of biofilm formation, 3×10^5^ cells were suspended in 1 ml of SC medium and incubated in each well of a 24-well polystyrene microplate at 37°C with 5% CO_2_. After 24 h, the wells were washed twice with sterile PBS. The degree of biofilm formation was determined using the XTT assay, as described above, except 0.125 mg/ml of XTT was used, and no menadione was added.

### Biofilm evaluation using scanning electron microscopy (SEM) and confocal scanning laser microscopy (CSLM)

The examination of *C*. *albicans* biofilm structure using SEM was performed as described previously [20]. Briefly, 3×10^5^ cells were grown on polystyrene coverslips (Thermanox plastic coverslip 174950, Thermo Scientific), which were placed in each well of a 24-well microplate containing 1 ml of SC medium. After biofilm formation, the coverslip was washed twice with PBS and fixed with 3.75% formaldehyde (in PBS) for 40 minutes. The post-fixed coverslip was treated with 1% osmium tetroxide for 5 minutes. After fixation, the samples were dehydrated with serial ethanol solutions (35% for 5 min; 50% for 5 min; 70% for 5 min; 80% for 5 min; 95% for 5 min; and 100% for 10 min twice), followed by overnight treatment with hexamethyldisilazane. The samples were then dried in a 60°C oven. Finally, the biofilms were examined and micrographs were collected using a SEM (Hitachi, S-4700, Type II).

To further examine the morphology and architecture of the biofilms, CSLM was used. The cells were grown on a polystyrene coverslip (Thermanox plastic coverslip 174950) in a 24-well microplate. After biofilm formation, the coverslip was washed with PBS and the cells were stained with 0.2 mg/ml of calcofluor (Sigma F-3543) for 1 h. The coverslip with the biofilm cells was placed on a regular microscope slide and covered with a regular microscope cover glass. A reinforcing ring was glued between the coverslip and the regular microscope cover glass to prevent the biofilm from becoming flattened. The morphology and architecture of the biofilms were examined using a Carl Zeiss LSM 510 confocal microscope with a 405 nm diode excitation laser.

## Results

### *C*. *albicans* orf19.5953 is a homolog of *S*. *cerevisiae* Sfp1

In our previous study, *C*. *albicans* orf19.5953 was identified as a promising transcription factor that may function in biofilm formation [21]. In the Candida Genome Database (http://www.candidagenome.org), *C*. *albicans* orf19.5953 is annotated as the homolog of *S*. *cerevisiae* Sfp1 and the functions of orf19.5953 are mostly uncharacterized.

In this work, to study the functions of Orf19.5953, we first aligned the amino acid sequences of *C*. *albicans* orf19.5953 and *S*. *cerevisiae* Sfp1 using Clustal W2 (Fig A in S1 File). The sequences of these two proteins share ~40% similarity and ~31% identity. In addition, both of these proteins contain two C2H2-type zinc finger domains in their C-terminal regions, which may be responsible for DNA binding (Fig A in S1 File).

**Fig 1:**
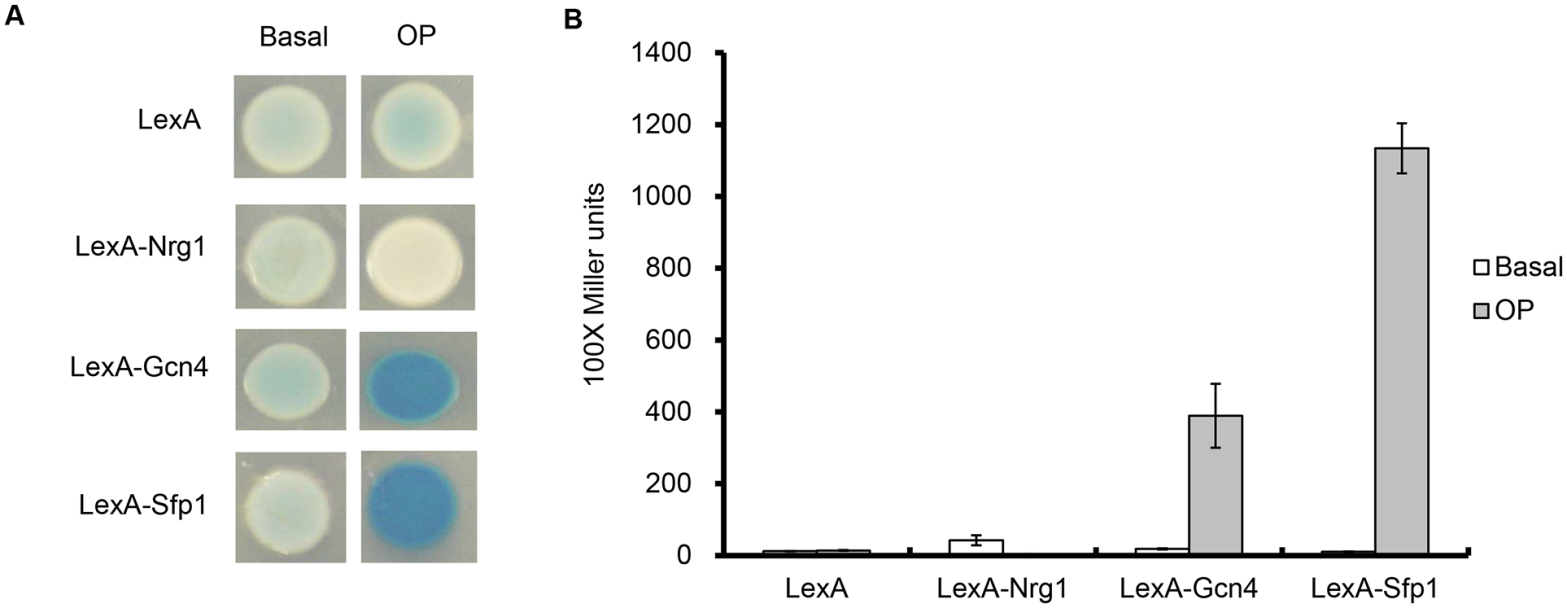
*C*. *albicans* Sfp1 functions as a transcriptional activator, as demonstrated by a one-hybrid analysis. Sfp1 was fused to LexA and regulated by the *ACT1* promoter. LexA-Sfp1 binds to the LexA operator (“OP”) upstream of the *lacZ* reporter gene. LacZ activity was measured using the X-Gal overlay assay (A) and the liquid β-galactosidase assay (B). A strain expressing only the LexA protein and a strain without the LexA operator (“basal”) upstream of the *lacZ* reporter gene were used as controls. The known activator Gcn4 and the repressor Nrg1 fused with LexA were used as positive and negative controls, respectively.

*S*. *cerevisiae* Sfp1 acts as an activator to regulate ribosome protein gene expression [33]. To investigate the role of *C*. *albicans* Sfp1 in transcriptional regulation, the one-hybrid assay [28] was performed. In the one-hybrid assay, both the X-gal overlay and the liquid β-galactosidase assay were performed. Strains that express LexA-Gcn4 and LexA-Nrg1 were used as controls for the activator and repressor, respectively. As shown in Fig 1, LexA-Sfp1 can strongly activate the expression of the *lacZ* reporter to the same extent as LexA-Gcn4. Moreover, in the liquid β-gal assay, LexA-Sfp1 activated the *lacZ* reporter gene even more strongly than LexA-Gcn4 (Fig 1). These results indicate that *C*. *albicans* Sfp1 is a transcriptional activator. Therefore, based on the sequence comparison and one-hybrid analysis, we refer to orf19.5953 as *C*. *albicans* Sfp1.

### *C*. *albicans* Sfp1 is involved in the regulation of ribosomal gene expression and is related to the TOR signaling pathway

To further reveal the functions of *C*. *albicans* Sfp1, we generated the *sfp1*-deleted and *SFP1*-reintegrated strains (Fig A in S1 File). The successful construction of these strains was verified by Southern blot analysis and real-time PCR (Fig A in S1 File). The growth rates of the WT, *sfp1*Δ/*sfp1*Δ and *SFP1*-reintegrated strains were then determined. As shown in Fig B in S1 File, *C*. *albicans sfp1*Δ/*sfp1*Δ mutants exhibited slower growth than the WT and *SFP1*-reintegrated strains, in both YPD and SC broth. A similar delay in cell growth was also observed after *SFP1* deletion in *S*. *cerevisiae* [34].

**Fig 2:**
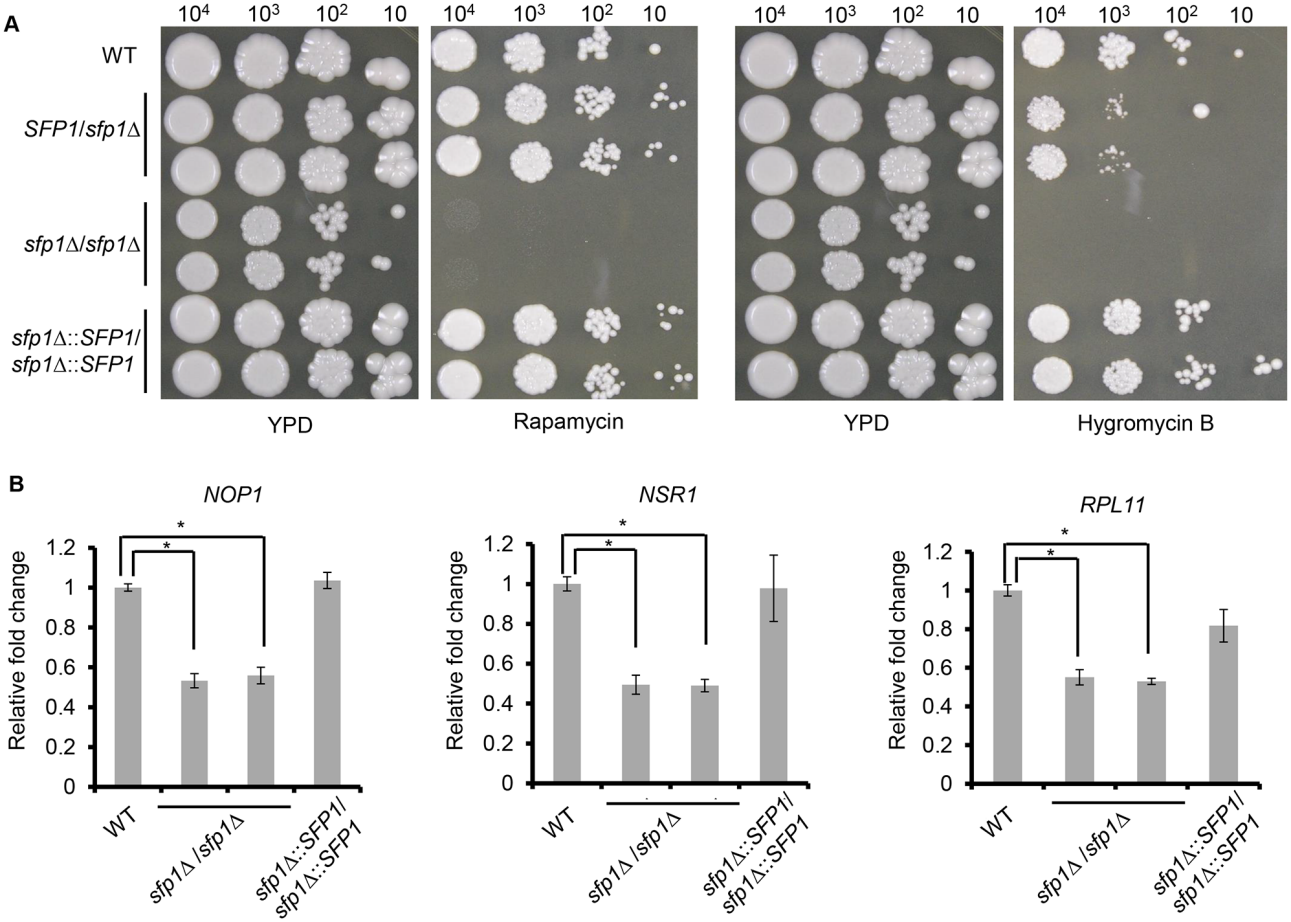
*C*. *albicans* Sfp1 regulates ribosomal gene expression and is related to the TOR signaling pathway. (A) Deletion of *SFP1* increases susceptibility to rapamycin and hygromycin B. The cells were ten-fold serially diluted and spotted onto YPD agar plates with or without rapamycin (25 ng/ml) and hygromycin B (400 μg/ml). The plates were incubated at 30°C for 5 days. (B) Deletion of *SFP1* affects ribosomal gene expression. The cells were grown in YPD medium overnight at 30°C, subcultured into fresh YPD medium and incubated until log phase (OD_600_ = 2). RNAs were isolated, cDNAs were generated and quantitative real-time PCR was performed. The expression levels of each gene are calculated as the mean ± standard deviation (SD) for three independent experiments. For each gene, the relative fold changes were displayed as the expression levels of an individual strain normalized to the WT strain (as 1). *, p < 0.05.

In addition, *S*. *cerevisiae* Sfp1p functions downstream of the target of rapamycin (TOR) kinase to regulate ribosomal protein (RP) and ribosome biogenesis (Ribi) gene transcription [33–36]. Moreover, the *S*. *cerevisiae sfp1*-deleted mutant is sensitive to hygromycin B, which is a drug that inhibits translation [37]. We hypothesize that *C*. *albicans* Sfp1 functions in a similar manner. To test this hypothesis, the WT, *sfp1*Δ/*SFP1*, *sfp1*Δ/*sfp1*Δ and *SFP1*-reintegrated strains were spotted onto agar plates with or without rapamycin. This drug is an inhibitor of the Tor1 kinase, which is the key enzyme of the *C*. *albicans* TOR signaling pathway. In Fig 2A, the *sfp1*Δ/*sfp1*Δ mutants were more sensitive to rapamycin than the WT, *sfp1*Δ/*SFP1* and *SFP1*-reintegrated strains, suggesting that *C*. *albicans* Sfp1 is related to the TOR signaling pathway. Moreover, the WT, *sfp1*Δ/*SFP1*, *sfp1*Δ/*sfp1*Δ and *SFP1*-reintegrated strains were spotted onto agar plates with or without hygromycin B. Similar to the findings obtained for *S*. *cerevisiae*, our results demonstrated that the *sfp1*Δ/*sfp1*Δ strains were much more sensitive to hygromycin B than the WT, *sfp1*Δ/*SFP1* and *SFP1*-reintegrated strains (Fig 2A).

To determine the role of *C*. *albicans* Sfp1 in the regulation of the RP and Ribi genes, RNAs were isolated from exponential phase cells and used for RT real-time qPCR. The expression of the RP (*RPL11*) and Ribi (*NOP1* and *NSR1*) genes was detected. As shown in Fig 2B, the expression of *NOP1*, *NSR1* and *RPL11* decreased in the *sfp1*Δ/*sfp1*Δ mutants compared to the WT and *SFP1*-reintegrated strains.

### Sfp1 affects cell adhesion and biofilm formation

**Fig 3:**
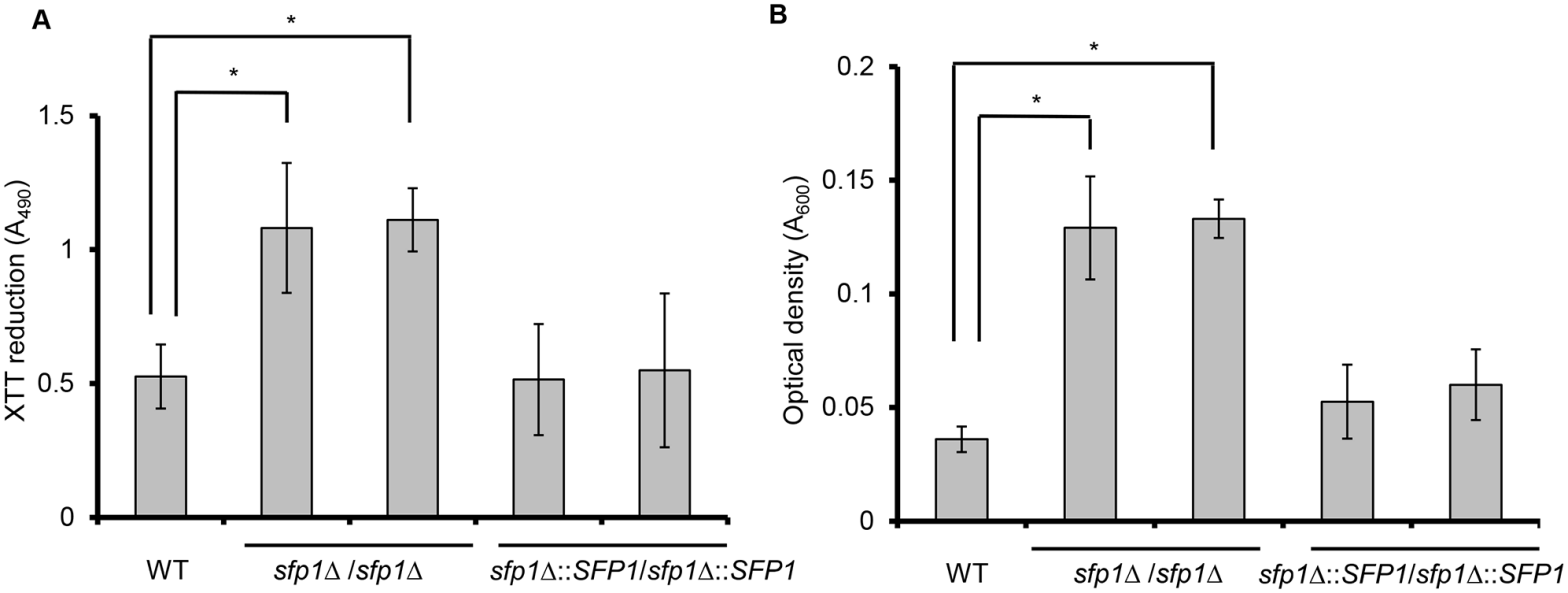
Deletion of *SFP1* enhances *C*. *albicans* adhesion. Cell adhesion was assessed in a 24-well polystyrene microplate in SC medium at 37°C with 5% CO_2_ for 1 h. The adherent cells were washed twice with PBS buffer. (A) Cell adhesion was determined using the XTT reduction assay. The results are presented as the mean ± SD from three independent experiments. *p < 0.05 for *sfp1*Δ/*sfp1*Δ vs. WT cells. (B) Cell adhesion was evaluated by measuring the density of the adherent cells. After washing, the adherent cells were scraped from the well, collected, and quantified by measuring the cell density (OD_600_). The results are presented as the mean ± SD from three independent experiments. *p < 0.05.

Using a computational approach, we hypothesized that Sfp1 is involved in biofilm formation [21]. Cell adhesion is the first step of biofilm formation. Therefore, the ability of WT, *sfp1*Δ/*sfp1*Δ and *SFP1*-reintegrated cells to adhere to a 24-well polystyrene microplate was compared using the XTT reduction assay. In metabolically active cells, the yellow tetrazolium salt XTT was reduced to a water-soluble orange-colored product whose absorbance can be measured at 490 nm [20]. In Fig 3A, the adhesion ability of the *sfp1*Δ/*sfp1*Δ strains was much higher than that of the WT and *SFP1*-reintegrated strains after 1 h incubation in SC medium. To further verify this finding, the adherent cells were scraped from the polystyrene surface and the optical density was measured at 600 nm. The results indicated that density of the adherent cells of the *sfp1*Δ/*sfp1*Δ strain was higher than that of the WT and *SFP1*-reintegrated strains (Fig 3B). Similar results were also obtained when cell adhesion was tested in YPD medium (Fig C in S1 File).

**Fig 4:**
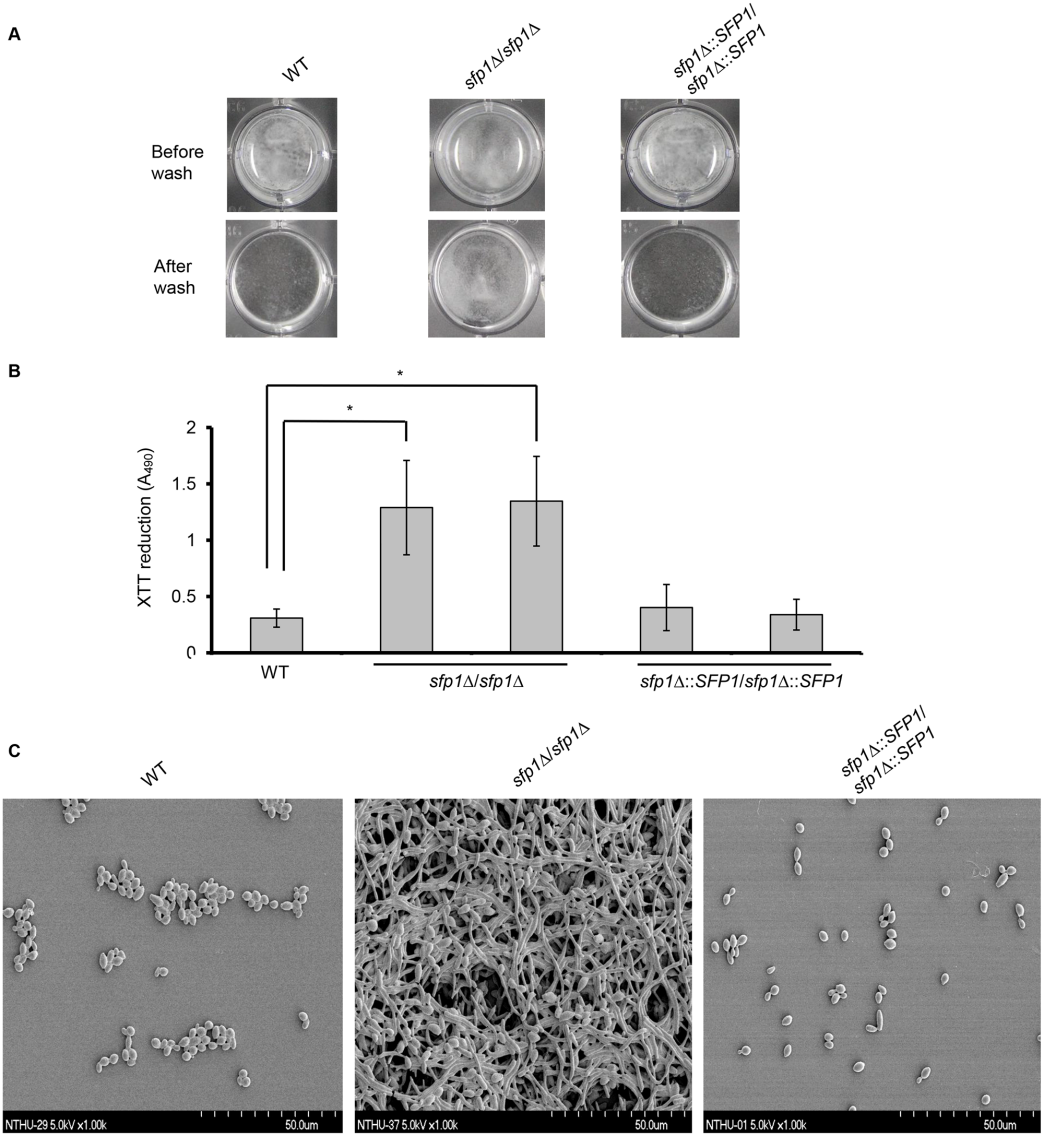
Deletion of *SFP1* enhances *C*. *albicans* biofilm formation. (A) Biofilms were formed on the surface of a 24-well polystyrene microplate in SC medium at 37°C with 5% CO_2_ for 24 h. (B) Biofilm formation was assessed using the XTT reduction assay. The results are presented as the mean ± SD from three independent experiments. *p < 0.05 for *sfp1*Δ/*sfp1*Δ vs. WT cells. (C) Biofilm structure was examined using scanning electron microscopy with a 1,000× magnification. Biofilm formation was carried out as described above, except the cells were grown on polystyrene coverslips for 24 h.

To further determine whether *sfp1*Δ/*sfp1*Δ strains can affect biofilm formation, cells were grown in SC medium in a 24-well polystyrene microplate for 24 h, and the mature biofilms in the plate were photographed and assayed using the XTT reduction method. The *sfp1*Δ/*sfp1*Δ strain exhibited much stronger biofilm formation than the WT and *SFP1*-reintegrated strains (Fig 4A and 4B). To further examine the structure of the formed biofilms, SEM was also used. The WT and *SFP1*-reintegrated strains exhibited a similar biofilm structure; the cells formed only a single layer of predominantly blastopore cells, which lacked a complex three-dimensional structure (Fig 4C). However, the structure of the *sfp1*Δ/*sfp1*Δ mutants differed considerably; the cells formed complex multilayered filamentous networks and internally embedded blastopore cells (Fig 4C). *C*. *albicans* biofilm formation was related to the growth medium used in a previous study [38]. Our finding that the deletion of *SFP1* can lead to increased biofilm formation was also observed in YPD medium (Fig C in S1 File). Collectively, our results indicate that Sfp1 negatively affects cell adhesion and biofilm in *C*. *albicans*.

### Sfp1 controls adhesin gene expression downstream of the Rhb1-TOR signaling pathway

**Fig 5:**
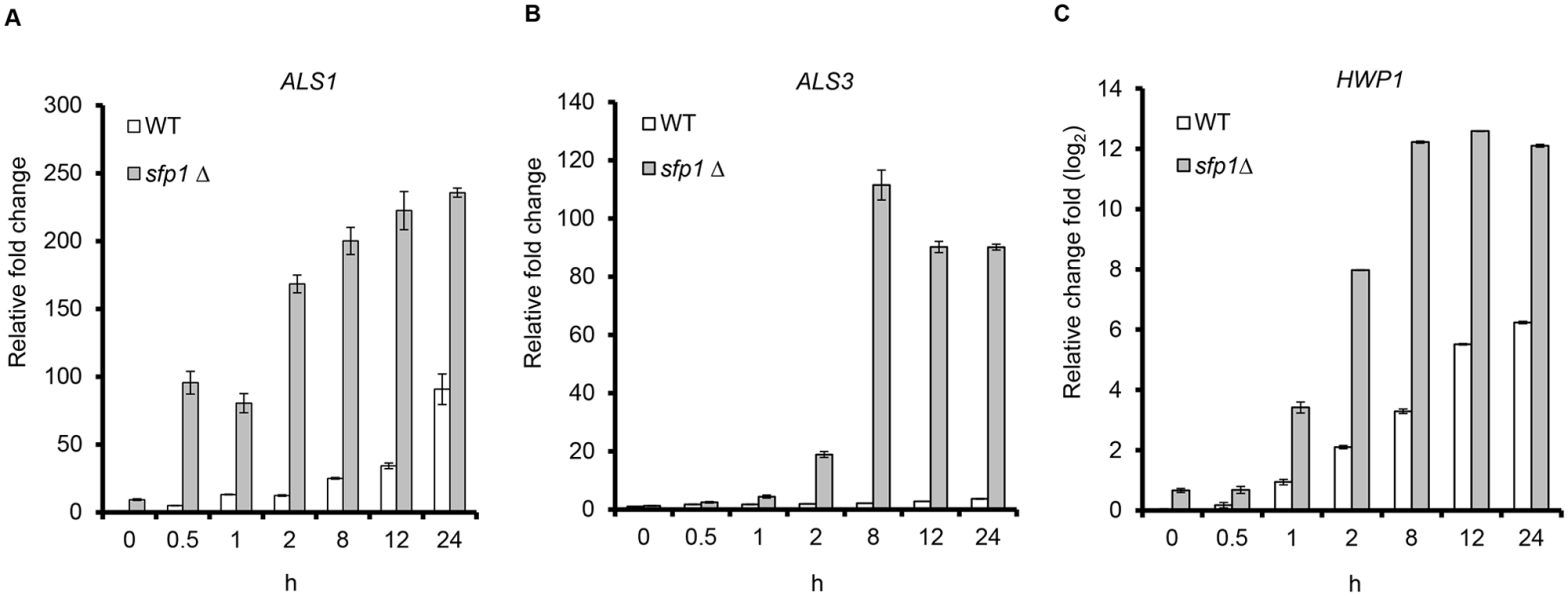
Deletion of *SFP1* enhances adhesin gene expression. The expression of adhesin genes was compared between the WT and *sfp1*Δ/*sfp1*Δ strains during biofilm formation in SC medium at 37°C with 5% CO_2_. Real-time qPCR was used to detect the expression of adhesin genes. *C*. *albicans PMA1* transcripts were used as an endogenous control. The results are presented as the mean ± SD from at least three independent experiments. For *HWP1* gene expression, the fold change was represented on a log2 scale.

The cell wall adhesin proteins Als1, Als3 and Hwp1 play critical roles in promoting *C*. *albicans* biofilm development [11,39]. The Tor1 kinase is involved in the regulation of adhesin gene expression [40]. Because the *sfp1*Δ/*sfp1*Δ mutants enhanced cell adhesion (Fig 3), we suspected that Sfp1 is involved in the regulation of the *ALS1*, *ALS3* and *HWP1* adhesin genes. RT real-time qPCR was performed, and dynamic patterns of gene expression were observed. For the *ALS1* gene, very low levels of expression were detected in both the WT and *sfp1*Δ/*sfp1*Δ strains at time 0, when the cells are planktonic and nonadherent (Fig 5A). During the cell adherence stage (approximately 0.5 to 1 h), the *ALS1* gene began to be induced in the *sfp1*Δ/*sfp1*Δ mutant compared to the WT strain (Fig 5A). Moreover, the expression of the *ALS1* gene was enhanced in the *sfp1*Δ/*sfp1*Δ mutant during the stages of biofilm development and maturation (approximately 2 to 24 h). A similar enhancement of *ALS3* and *HWP1* gene expression was also detected in the *sfp1*Δ/*sfp1*Δ mutants compared to the WT strain during the development and maturation of *C*. *albicans* biofilms (Fig 5B and 5C).

**Fig 6:**
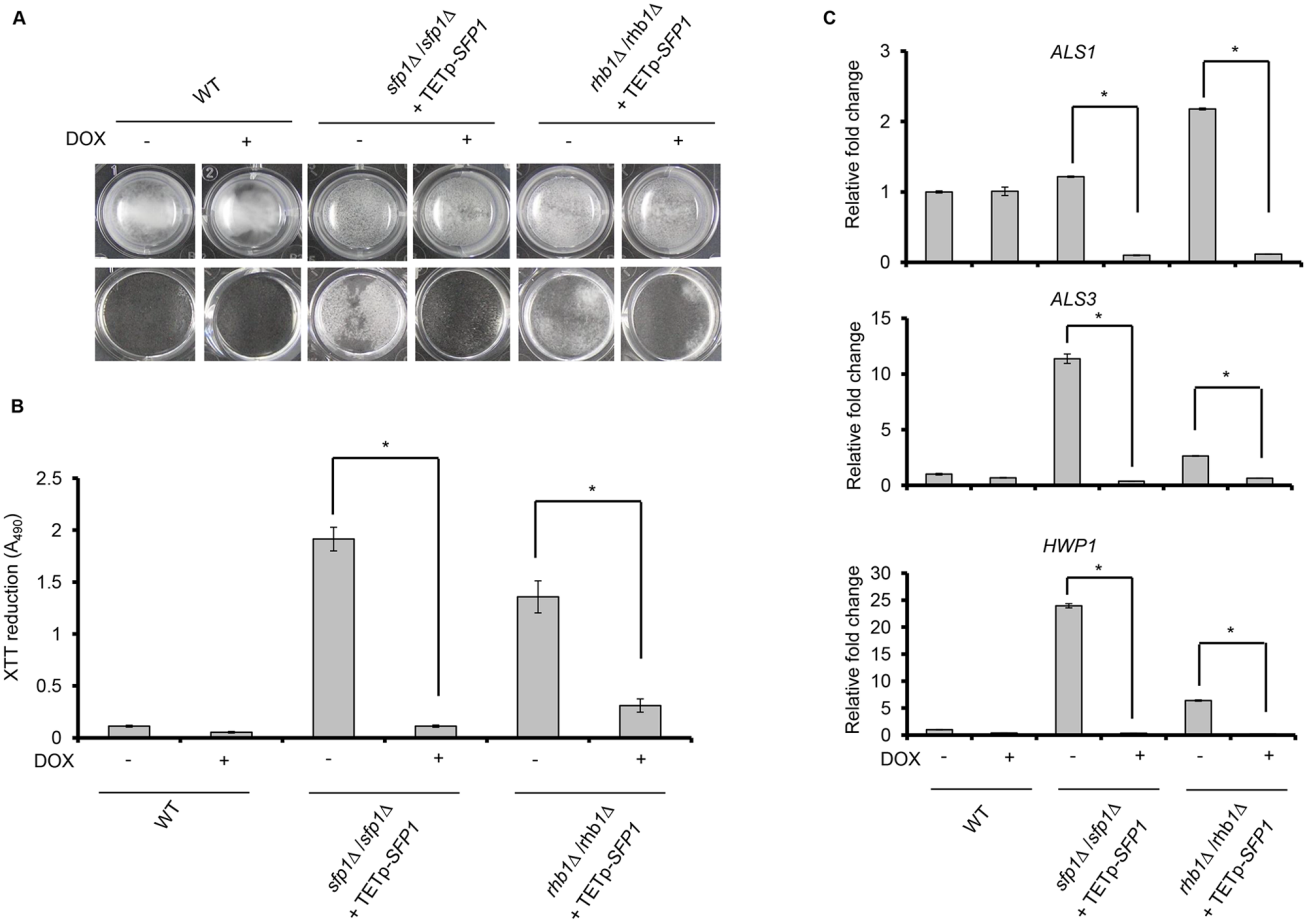
Rhb1 is involved in Sfp1-mediated regulation of biofilm formation. Biofilm formation was compared between the *sfp1*Δ/*sfp1*Δ strain containing TETp-*SFP1* and the *rhb1*Δ/*rhb1*Δ strain containing TETp-*SFP1*. Biofilms were formed for 24 h in SC medium at 37°C with 5% CO_2_, with or without 50 μg/ml of doxycycline (DOX). Doxycycline induces the overexpression of the *SFP1* gene, which is under the control of a tetracycline-inducible promoter (TETp). (A) Biofilms were formed on the surface of a 24-well polystyrene microplate in SC medium at 37°C with 5% CO_2_ for 24 h. Biofilm cells are shown before washing (top) and after washing (bottom). Representative images from three independent experiments with similar results are shown. (B) Biofilm formation was measured using the XTT reduction assay. The results are presented as the mean ± SD from three independent experiments. *p < 0.05 for cells without DOX treatment vs. DOX-treated cells. (C) The expression of adhesin genes was compared in the presence and absence of *SFP1* overexpression in the *SFP1*- or *RHB1*-deleted background. Real-time qPCR was performed, and *C*. *albicans PMA1* transcripts were used as an endogenous control. The results are presented as the mean ± SD from at least three independent experiments. *p < 0.05 for cells without DOX treatment vs. cells with DOX treatment.

The association of Sfp1 with the TOR signaling pathway was proposed based on Fig 2. Interestingly, our previous studies linked the small GTPase Rhb1 to the Tor1 kinase in the control of filamentation and the secreted aspartyl protease 2 (Sap2), which are both important virulence factors of *C*. *albicans* [41,42]. Therefore, this finding suggests a possible connection between Sfp1 and Rhb1. To test this hypothesis, biofilm formation was compared among the *sfp1*Δ/*sfp1*Δ, *rhb1*Δ/*rhb1*Δ and *sfp1*Δ/*sfp1*/Δ*rhb1*Δ/*rhb1*Δ strains. Fig D in S1 File showed that all the *sfp1*Δ/*sfp1*Δ, *rhb1*Δ/*rhb1*Δ and *sfp1*Δ/*sfp1*/Δ*rhb1*Δ/*rhb1*Δ formed a robust biofilm in SC medium compared to the controls (WT, *SFP1*-reintegrated and *RHB1*-reintegrated strains). Therefore, these results were not helpful to detect epistatic relationship between *SFP1* and *RHB1* genes. Alternatively, we compared the strains with *SFP1* overexpression in the *sfp1*Δ/*sfp1*Δ or *rhb1*Δ/*rhb1*Δ background. In the presence of the tetracycline derivative doxycycline a gene can be overexpressed under the control of a tetracycline-inducible promoter [26]. As shown in Fig 6A and 6B, the biofilm formation ability of the WT strain was not affected in the presence or absence of doxycycline. The *sfp1*Δ/*sfp1*Δ mutant exhibited enhanced biofilm formation, whereas the overexpression of *SFP1* in the *sfp1*Δ/*sfp1*Δ background significantly decreased biofilm formation. Similar to the *sfp1*Δ/*sfp1*Δ mutant, enhanced biofilm formation was observed for the *rhb1*Δ/*rhb1*Δ strain without doxycycline, but doxycycline-induced *SFP1* overexpression in the *rhb1*Δ/*rhb1*Δ background significantly suppressed biofilm formation. Finally, reduced expression of adhesin genes was also detected after *SFP1* overexpression in the *sfp1*Δ/*sfp1*Δ and *rhb1*Δ/*rhb1*Δ strains (Fig 6C). Based on the results of this study and other studies [40], Sfp1 appears to function downstream of the Rhb1-Tor1 signaling pathway.

### Sfp1 may regulate adhesin genes and biofilm formation through Bcr1 and Efg1

**Fig 7:**
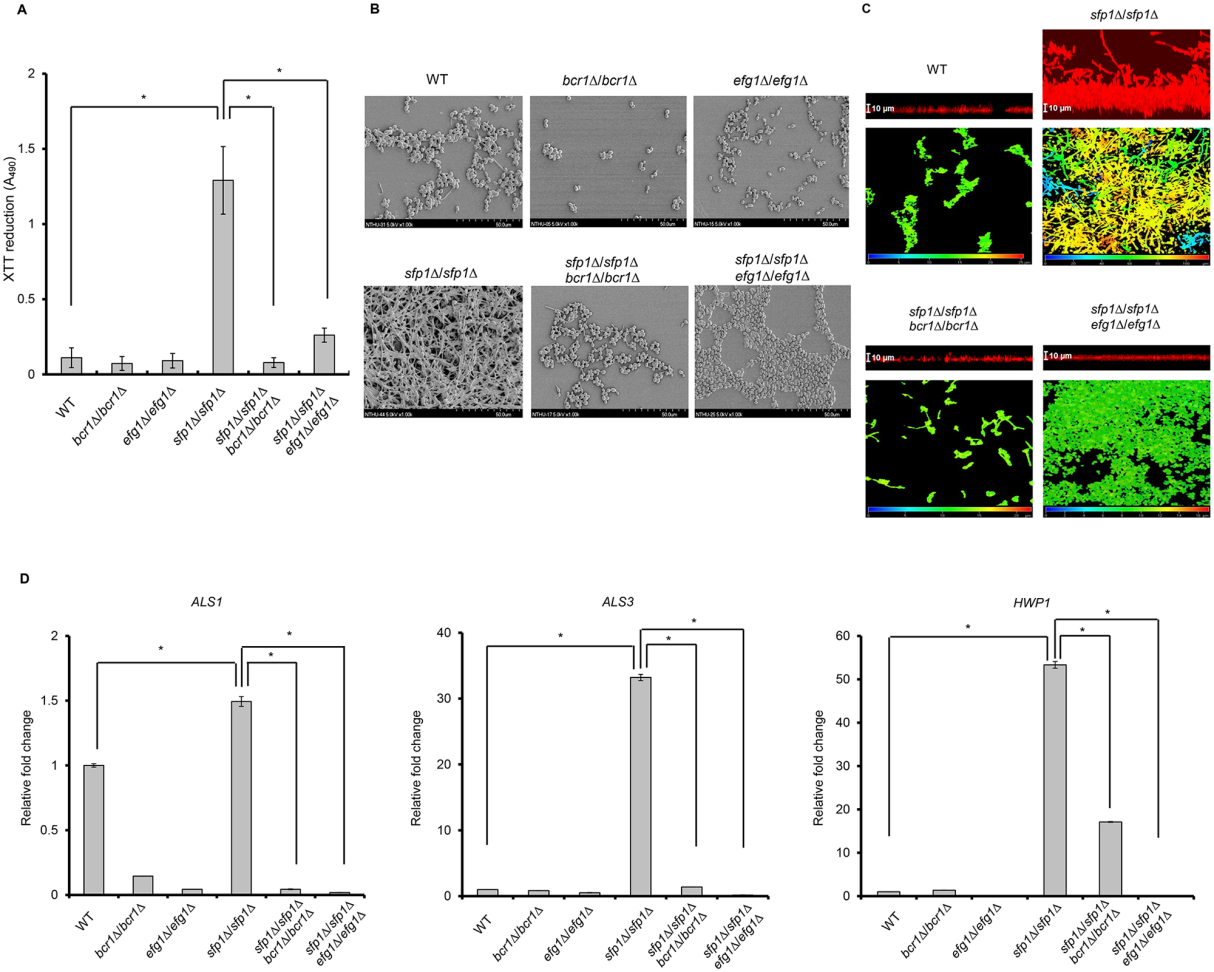
Sfp1 regulates biofilm formation through Bcr1 and Efg1. (A) Biofilm formation was assessed using the XTT reduction assay. The results are presented as the mean ± SD from three independent experiments. *p < 0.05 for *sfp1*Δ/*sfp1*Δ vs. WT, *bcr1*Δ/*bcr1*Δ/*sfp1*Δ/*sfp1*Δ or *efg1*Δ/*efg1*Δ/*sfp1*Δ/*sfp1*Δ mutant cells. (B) The biofilm structure was examined using scanning electron microscopy. Pictures were taken at a 1,000× magnification. The cells were grown on polystyrene coverslips for 24 h to form biofilms. (C) The biofilm structure was examined using confocal scanning laser microscopy (CSLM). Biofilms were formed on the polystyrene coverslips in SC medium at 37°C with 5% CO_2_ for 24 h. After washing, the cells were stained with 0.2 mg/ml of calcofluor (Sigma F-3543, “Fluorescent brightener 28”) for CSLM visualization. Pictures were taken at a 400× magnification. (D) The expression of adhesin genes in biofilm cells was detected using RT real-time qPCR. *PMA1* transcripts were used as an endogenous control. The results are presented as the mean ± SD from at least two independent experiments. *p < 0.05 for *sfp1*Δ/*sfp1*Δ vs. WT, *bcr1*Δ/*bcr1*Δ/*sfp1*Δ/*sfp1*Δ or *efg1*Δ/*efg1*Δ/*sfp1*Δ/*sfp1*Δ mutant cells.

In addition to Tor1, the transcription factors Efg1 and Bcr1 are also known to control the *ALS1*, *ALS3* and *HWP1* genes and play a role in the activation of *C*. *albicans* biofilm formation [19,43–45]. Because Sfp1 can regulate the expression of the *ALS1*, *ALS3* and *HWP1* genes (Fig 5), the relationship between Sfp1, Efg1 and Bcr1 in adhesin gene regulation and biofilm formation is of interest. To address this question, we constructed *bcr1*Δ/*bcr1*Δ/*sfp1*Δ/*sfp1*Δ and *efg1*Δ/*efg1*Δ/*sfp1*Δ/*sfp1*Δ double mutants. Biofilm formation determined by the XTT reduction method showed that the *sfp1*Δ/*sfp1*Δ mutant significantly enhanced biofilm formation compared to the controls (WT, *bcr1*Δ/*bcr1*Δ and *efg1*Δ/*efg1*Δ strains). Interestingly, both the *bcr1*Δ/*bcr1*Δ/*sfp1*Δ/*sfp1*Δ and *efg1*Δ/*efg1*Δ/*sfp1*Δ/*sfp1*Δ double deletion mutants exhibited poor biofilm-forming abilities similar to that of the controls (Fig 7A). Moreover, SEM examination demonstrated that the *sfp1*Δ/*sfp1*Δ mutant formed a complex multilayered biofilm, while both the *bcr1*Δ/*bcr1*Δ and *efg1*Δ/*efg1*Δ mutants formed only a single layer of cells similar to that of WT (Fig 7B). However, the *bcr1*Δ/*bcr1*Δ/*sfp1*Δ/*sfp1*Δ and *efg1*Δ/*efg1*Δ/*sfp1*Δ/*sfp1*Δ double deletion mutants also formed a single layer of structure containing predominantly blastopore cells (Fig 7B). The biofilm structures were also examined using CSLM, and the biofilm of the *sfp1*Δ/*sfp1*Δ mutant exhibited a complex multilayered structure with a thickness of ~80 μm. However, the *bcr1*Δ/*bcr1*Δ/*sfp1*Δ/*sfp1*Δ and *efg1*Δ/*efg1*Δ/*sfp1*Δ/*sfp1*Δ double mutants exhibited reduced biofilm formation and only formed a very thin layer (~10 μm) of cell structure (Fig 7C). Because both Bcr1 and Efg1 are known to promote biofilm formation [44,46], our results suggest that Sfp1 suppresses biofilm formation via the negative regulation of Bcr1 and Efg1.

To further reveal the relationships among Bcr1, Efg1 and Sfp1, the expression of adhesin genes was also examined using RT real-time qPCR. The *ALS1*, *ALS3* and *HWP1* genes were highly expressed in the *sfp1*Δ/*sfp1*Δ mutant, in agreement with the real-time qPCR analysis in Fig 5. However, the deletion of *BCR1* or *EFG1* in the *sfp1*Δ/*sfp1*Δ background significantly reduced the expression levels of all three adhesin genes (Fig 7D). Based on the biofilm structure and adhesin gene expression, Bcr1 and Efg1 appear to function as downstream effectors of Sfp1.

## Discussion

The use of indwelling medical devices has become routine in the clinical setting. *C*. *albicans* easily adheres to and forms a biofilm on indwelling medical devices. Such biofilms require subsequent surgical removal and replacement of the infective devices. *C*. *albicans* cells can also adhere to and form a biofilm on mucosal surfaces, leading to resistance to antifungal agents and the initiation of infections. Therefore, *C*. *albicans* biofilms are a serious public health problem. Many recent studies focused on the regulation of biofilm formation. One study that was performed by Nobile *et al*. identified a transcriptional network that controls biofilm development [18]. This study combined ‘‘classical” genetics, genome-wide approaches, and RNA deep sequencing technology to comprehensively map the transcriptional circuitry that controls biofilm formation in *C*. *albicans*. In addition, this study also described a master circuit of six transcription regulators, including Bcr1, Tec1, Efg1, Ndt80, Rob1, and Brg1, that controls approximately 1,000 target genes and biofilm formation in vitro and in two animal models [18]. This master circuit led to many new predictions about the genes involved in biofilm formation, and some of those predicted genes have been confirmed to play roles in biofilm development. Another study that was performed by Wang *et al*. computationally screened for potential transcription factors that could regulate *C*. *albicans* biofilm [21]. In that study, gene expression profiles and ChIP-chip were used to establish biofilm and planktonic gene regulatory networks. The two networks were subsequently compared, and the relevance value was calculated to identify potential transcription factors related to biofilm formation. Among the identified candidates, the relevance value of Sfp1 (Orf19.5943) was the highest and that gene was considered the most relevant for biofilm formation. The aim of this study was to examine the functions of Sfp1, particularly the role of Sfp1 in biofilm formation.

Previous studies demonstrated that the growth medium may affect *C*. *albicans* biofilm formation under laboratory conditions [32,38,47,48]. In this study, we tested several growth media for biofilm formation, including Lee’s, Spider, SC and YPD media (Fig 4, Fig C in S1 File and Fig F in S1 File). Among the tested media, although the cells showed a growth delay (Fig B in S1 File), both the WT and *sfp1*Δ/*sfp1*Δ strains formed biofilms in the Lee’s and Spider media (Fig F in S1 File). However, the WT and *sfp1*Δ/*sfp1*Δ strains exhibited significant differences in biofilm formation in SC and YPD media (Fig 4 and Fig C in S1 File). Particularly, the WT and *SFP1*-reintegrated strains produced only a rudimentary biofilm in the SC medium, but the *sfp1*Δ/*sfp1*Δ mutant could form a robust biofilm with abundant filamentous cells (Fig 4). Mutant cells that are fixed in either the hyphal or yeast form can only develop into a rudimentary biofilm that is not as stable as a normal mature biofilm that contains both cell morphologies [49]. The *sfp1*Δ/*sfp1*Δ mutants exhibited highly enhanced biofilm formation compared to the WT strain, suggesting that Sfp1 negatively regulates biofilm formation under these experimental conditions. To reveal the mechanisms via which Sfp1 affects biofilm formation, we thus used the SC medium for most of our experiments.

Adhesion is the first key step for biofilm formation. Cell adhesion may be mediated by non-specific factors, including hydrophobicity and electrostatic forces of the cell surface, or by specific adhesins on the surface of *C*. *albicans*. Fig 3 demonstrated that the *sfp1*Δ/*sfp1*Δ mutants exhibit significantly enhanced cell adhesion in polystyrene microplates, indicating that Sfp1 suppresses cell adhesion. The *SFP1* deletion may somehow alter the *C*. *albicans* cell wall architecture and composition, leading to changes in the non-specific properties of the cell surface. Coincidentally, the *sfp1*Δ/*sfp1*Δ mutants were resistant to the cell wall disrupting agents Congo red and calcofluor white and hyper-resistant to zymolyase, which has strong lytic activity against cell wall β-1,3-glucan (data not shown). These results implied that the *SFP1* gene deletion alters cell wall structure and composition, leading to changes in adhesion-related cell surface properties.

However, other evidence suggests that Sfp1 can also regulate cell adhesion by regulating the expression of cell wall adhesin genes. The deletion of *SFP1* increased *ALS1* and *HWP1* adhesin gene expression 0.5 h and 1 h after the cells adhered to the substrate (Fig 5). The easy detection of the *ALS1* gene during the early adhesion stage of *C*. *albicans* yeast cells was reported previously [50]. Hwp1 is also required for covalent attachment to host epithelial cells and virulence [23]. Strains that lack either *ALS1* or *HWP1* can lose their abilities to attach to an abiotic surface and form a biofilm [18,51]. Moreover, *ALS3* and *HWP1* are expressed primarily in hyphal cells [50,52]. In Fig 5, the expression of *ALS3* and *HWP1* in the *sfp1*Δ/*sfp1*Δ strain began to be induced after cell attachment for 2 h, which is when the cells began to form germ tubes (Fig E in S1 File). Moreover, the Als1, Als3 and Hwp1 adhesins also play a complementary role in biofilm formation. A previous study demonstrated that both the *hwp1*Δ/*hwp1*Δ mutant and the *als1*Δ/*als1*Δ/*als3*Δ/*als3*Δ double mutant strain are defective in biofilm formation; however, a mixture of these two strains can form robust biofilms both in vitro and in vivo [39]. In addition, the *hwp1*Δ/*hwp1*Δ mutant produced a biofilm with significantly less biomass than the WT strain [39]. *C*. *albicans* mutants that lack Als3 produce scarce, defective biofilms on catheter material in vitro [46]. Although the functions of Als1 and Als3 in biofilm formation are somewhat overlapping, Als1 may not play as pivotal a role as Als3 [39]. An *als1*Δ/*als1*Δ mutant had only a partial defect in biofilm formation [18], whereas the *als3* mutant displayed a severe defect in biofilm formation [46]. Following the early adhesion stage, the expression of all three adhesin genes increased gradually in the *sfp1*Δ/*sfp1*Δ strain (Fig 5). Together, these results suggest that *SFP1* deletion can enhance biofilm development, possibly through the derepression of adhesin gene expression.

In addition to Sfp1, the transcription factors Bcr1 and Efg1 can also regulate adhesin gene expression [19,43,46,53,54] in a manner dependent on the Tor1 kinase [40]. Moreover, the *bcr1*Δ/*bcr1*Δ mutant failed to produce a mature biofilm and instead formed only a rudimentary biofilm composed of yeast and hyphal cells [19]. The *efg1*Δ/*efg1*Δ mutant also fails to form biofilms and instead generates a sparse monolayer of yeast cells without hyphae [44,55]. Efg1 was also demonstrated to be involved in both normoxic and hypoxic biofilm formation [45]. Interestingly, we found that the *sfp1*Δ/*sfp1*Δ strain was hypersensitive to rapamycin (Fig 2A) and rapamycin enhanced biofilm formation in the WT strain, leading to the formation of biofilms that were similar to those of the *sfp1*Δ/*sfp1*Δ mutant without rapamycin treatment (Fig G in S1 File). Taken together, these results and the fact that Sfp1 also regulates adhesin genes and affects cell adhesion and biofilm formation (Figs 3–5) suggest the existence of relationships among Sfp1, Efg1 and Bcr1. The *bcr1*Δ/*bcr1*Δ/*sfp1*Δ/*sfp1*Δ double mutant exhibited dramatically reduced adhesin gene expression in comparison to the *sfp1*Δ/*sfp1*Δ mutant (Fig 7D). More importantly, the biofilm of the *bcr1*Δ/*bcr1*Δ/*sfp1*Δ/*sfp1*Δ double mutant contained fewer adherent cells after a 24 h incubation than the biofilm of the *sfp1*Δ/*sfp1*Δ mutant, which contained a large number of attached cells (Fig 7A–7C). In addition, the *bcr1*Δ/*bcr1*Δ/*sfp1*Δ/*sfp1*Δ mutant had nearly no filamentous cells in the biofilm, in contrast to the *sfp1*Δ/*sfp1*Δ mutant (Fig 7B). Although Bcr1 is not required for hyphal growth, this transcription factor is required for the activation of several hyphal-specific genes, including *ALS3* and *HWP1*. Our result is consistent with a previous biofilm assay, in which the WT strain formed a biofilm that consisted of abundant hyphal cells but the *bcr1*Δ/*bcr1*Δ strain generated a thin rudimentary biofilm that was comprised largely of yeast form cells [46]. Thus, our results suggested that Bcr1 appeared to induce adhesin expression in the absence of the *SFP1* gene during biofilm formation in SC medium. In addition, the *sfp1*Δ/*sfp1*Δ/*efg1*Δ/*efg1*Δ exhibited reduced biofilm formation and was defective in filamentous growth compared with the *sfp1*Δ/*sfp1*Δ strain (Fig 7B). Moreover, the *efg1*Δ/*efg1*Δ/*sfp1*Δ/*sfp1*Δ double mutant also exhibited dramatically reduced adhesin gene expression in comparison to the *sfp1*Δ/*sfp1*Δ mutant (Fig 7D).

**Fig 8:**
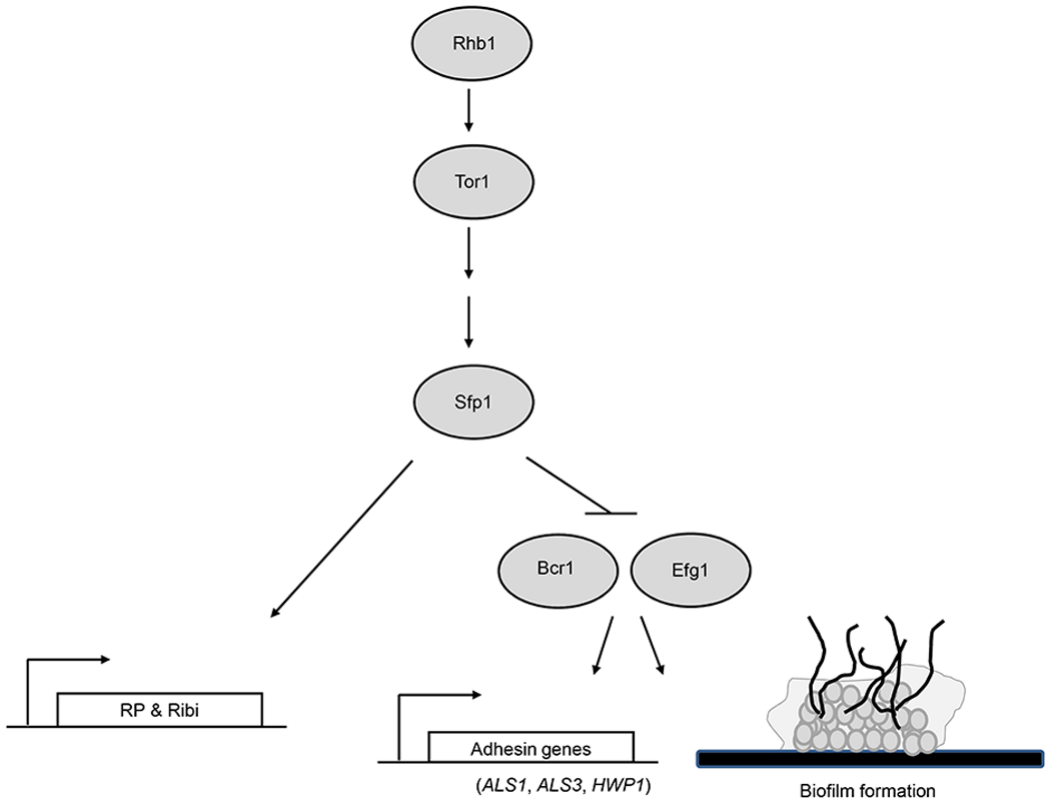
A simple model of the role of Sfp1 in the regulation of ribosomal gene expression and biofilm formation. Sfp1 is related to the Tor1 signaling pathway and plays a role in the induction of RP and Ribi gene expression. Moreover, during the regulation of adhesin gene expression and biofilm formation, the small GTPase Rhb1 coordinates with Tor1 to function upstream of Sfp1, while the transcription factors Bcr1 and Efg1 function downstream of Sfp1.

Taken together, these findings suggest a simple model for the functions of *C*. *albicans* Sfp1, as presented in Fig 8. Similar to *S*. *cerevisiae* Sfp1, *C*. *albicans* Sfp1 functions as an activator to regulate ribosomal gene expression. However, we further expand the function of Sfp1 to include a role in *C*. *albicans* biofilm formation, downstream of the Rhb1-Tor1 signaling pathway. Moreover, the functions of Sfp1 appear to be mediated by the negative regulation of the transcription factors Bcr1 and Efg1, which activate adhesin genes and promote biofilm formation (Fig 8). Although this study provides some new insights into the regulation of *C*. *albicans* biofilm formation, many questions must still be addressed. For example, the relationships among Sfp1, Ndt80 and Rob1 during biofilm formation remain unclear. Moreover, most of the transcription factors that have been identified to date are related to the activation of biofilm formation. In this study, we demonstrate that Sfp1 plays a negative role in biofilm formation. The identification of additional transcriptional repressors of biofilm formation is important. These studies will allow us to understand the complex interplay of negative and positive regulation in the context of biofilm formation.

## Supporting Information

S1 File Supplementary Tables and Figures. (DOCX)
