# Supplementary material for: Role of SFP1 in the Regulation of Candida albicans Biofilm Formation
Source: PLoS One. 2015 Jun 18;10(6):e0129903. doi: 10.1371/journal.pone.0129903 (PMC4472802; doi:10.1371/journal.pone.0129903)
Supplement: S1 File — (DOCX) [file pone.0129903.s001.docx]

**Supporting Information**

Supplemental Table A. Strains used in this study.

Supplemental Table B. Oligonucleotides used in this study.

Supplemental Figure A. Construction of the *sfp1*∆/*sfp1*△ and *SFP1*-reintegrated strains.

Supplemental Figure B. *C. albicans* *SFP1*-deletion strains exhibit a growth delay compared with the wild type and *SFP1*-reintegrated strains.

Supplemental Figure C. Deletion of *SFP1* enhances *C. albicans* adhesion and biofilm formation in YPD medium.

Supplemental Figure D. Deletion of *RHB1* enhances *C. albicans* biofilm formation.

Supplemental Figure E. Cell morphology during the process of biofilm formation in the WT and *sfp1*∆/*sfp1*△ strains.

Supplemental Figure F. WT, *sfp1*Δ/*sfp1*Δ and *SFP1*-reintegrated strains all could form biofilm in Lee’s and Spider media.

Supplemental Figure G. Inhibition of Tor1 enhances *C. albicans* biofilm formation.

**Supplemental Table A. Strains used in this study.**

| **Strain name** | **Genotype** | **Source** |
| --- | --- | --- |
| SC5314 | Wild type | [1] |
| *SFP1* mutants  *sfp1*D6-17 | *sfp1*∆::*FRT*/ *sfp1*∆::*FRT* | This study |
| *sfp1*D1-3 | *sfp1*∆::*FRT*/ *sfp1*∆::*FRT* | This study |
| *SFP1*RD6-17 | *sfp1∆*::*SFP1-FRT/ sfp1∆*::*SFP1-FRT* | This study |
| *SFP1*RD1-3 | *sfp1∆*::*SFP1-FRT/ sfp1∆*::*SFP1-FRT* | This study |
| Yeast one-hybrid assay  COP1  CCR1  OC1  CC1  OG21  CG21  ON18  CN18  COP-LexASfp1  CCR-LexASfp1  *BCR1 EFG1* mutants  *sfp1*D*bcr1*D  *sfp1*D*efg1*D | *CAI8 ade2*::*hisG/ade2*::*hisG*::[pCR-OPlacZ]  *CAI8 ade2*::*hisG/ade2*::*hisG*::[pCR-lacZ]  COP1 *RPS1/RPS1*::[CIp-LexA-F1]  CCR1 *RPS1/RPS1*::[CIp-LexA-F1]  COP1 *RPS1/RPS1*::[CIp-LexA-F-Gcn4-21]  CCR1 *RPS1/RPS1*::[CIp-LexA-F-Gcn4-21]  COP1 *RPS1/RPS1*::[CIplexA-F-CaNrg1-18]  CCR1 *RPS1/RPS1*::[CIplexA-F-CaNrg1-18]  COP1 *RPS1*/*RPS1*::[CIp-LexA-F-Sfp1]  CCR1 *RPS1*/*RPS1*::[CIp-LexA-F-Sfp1]  *sfp1*∆::*FRT*/ *sfp1*∆::*FRT* *bcr1*∆::*FRT*/ *bcr1*∆::*FRT*  *sfp1*∆::*FRT*/ *sfp1*∆::*FRT* *efg1*∆::*FRT*/ *efg1*∆::*FRT* | [2]  [2]  [2]  [2]  [2]  [2]  [2]  [2]  This study  This study  This study  This study |
| SFP1 overexpression  *sfp1*D+TETp-SFP1  *rhb1*D+TETp-SFP1 | *sfp1*∆*/sfp1*∆ *ADH1/adh1::*Ptet*-SFP1*  *rhb1*∆*/rhb1∆* *ADH1/adh1::*Ptet*-SFP1* | This study  This study |

1. Gillum AM, Tsay EY, Kirsch DR. Isolation of the *Candida albicans* gene for orotidine-5'-phosphate decarboxylase by complementation of *S. cerevisiae* ura3 and *E. coli* pyrF mutations. Mol Gen Genet. 1984; 198: 179-182.

2. Hsu PC, Yang CY, Lan CY. *Candida albicans* Hap43 is a repressor induced under low-iron conditions and is essential for iron-responsive transcriptional regulation and virulence. Eukaryot Cell 2011; 10: 207-225.

**Supplemental Table B. Oligonucleotides used in this study.**

| **Name** | **Description** | **Sequence (5’ to 3’)^a^** |
| --- | --- | --- |
| *SFP1* deletion and reintegration  CaSFP1uF-KpnI | 5’ flanking region of *SFP1*, forward primer | GTGAGGTACCAATAAGTGTCGCATCCAAAC |
| CaSFP1uR-XhoI | 5’ flanking region of *SFP1*, reverse primer | GACCCTCGAGCCTGATGAGCCTGTATTTTC |
| CaSFP1dF-SacII | 3’ flanking region of *SFP1*, forward primer | ACATCCGCGGTGGGCATACCACTCATTAAA |
| CaSFP1dR-SacI | 3’ flanking region of *SFP1*, reverse primer | GCAGGAGCTCAGAAGACCAAGTAGGCATCA |
| CaSFP1-2R-XhoI | For *SFP1* ORF, reverse primer, used with CaSFP1uF-KpnI | TCCGCTCGAGTTAATGAGTGGTATGCCCAC |
| Yeast one-hybrid assay  SFP1 one-hybrid-1  SFP1 one-hybrid-2  Tetracycline-induced overexpression  SFP1 Tet-on SalI-F1    SFP1 Tet-on BglII-R2  *BCR1* and *EFG1* deletion | For pCIp-LexA-F-SFP1 construction  For pCIp-LexA-F-SFP1 construction  For construction of pNIM1 containing Ptet-SFP1-SAT1 cassette  For construction of pNIM1 containing Ptet-SFP1-SAT1 cassette | GGTCCACGCGTGGTGGAGGTCCAGGTGGAATGTTTAATACCAAGATATTTGAAA  GCCCGCCTGCAGTCATTAATGAGTGGTATGCCCACGA  CCGGGTCGACATGTTTAATACCAAGATATT  CCGCAGATCTTTAATGAGTGGTATGCCCAC |
| BCR1 UR F1-KpnI    BCR1 UR R2-XhoI | 5’ flanking region of *BCR1*, forward primer  5’ flanking region of *BCR1*, reverse primer | GTGAGGTACCCGAACACACGTTCCCTGTTA  GAAACTCGAGTTTCAAAGTTGGCAGTGACG |
| BCR1 DR F1-SacII | 3’ flanking region of *BCR1*, forward primer | ACATCCGCGGTGGGGTGACGACAGTGTTTA |
| BCR1 DR R2-SacI | 3’ flanking region of *BCR1*, reverse primer | GCAGGAGCTCGCAGGAGGAGAAGAAGGAAGA |
| EFG1-UR-F-KpnI | 5’ flanking region of *EFG1*, forward primer | GTGAGGTACCTCATTGCACTAACCCGAACA |
| EFG1-UR-R-XhoI | 5’ flanking region of *EFG1*, reverse primer | GATTCTCGAGGGGTTAAGGGTTGGTTGGTT |
| EFG1-DR-F-SacII | 3’ flanking region of *EFG1*, forward primer | ACATCCGCGGCAGCTAGCACTGTTGCCAAA |
| EFG1-DR-R-SacI  Real time PCR  ALS1-qF-1  ALS1-qR-2  ALS3-qF-1  ALS3-qR-2  HWP1-qF-1  HWP1-qR-2  SFP1-qF1  SFP1-qR2  PMA1-qF-1  PMA1-qR-2  RT-PCR  ALS1-1  ALS1-2  ALS3-1  ALS3-2  HWP1-1  HWP1-2  PMA1-1  PMA1-2 | 3’ flanking region of *EFG1*, reverse primer  Forward primer  Reverse primer  Forward primer  Reverse primer  Forward primer  Reverse primer  Forward primer  Reverse primer  Forward primer  Reverse primer  Forward primer  Reverse primer  Forward primer  Reverse primer  Forward primer  Reverse primer  Forward primer  Reverse primer | GCAGGAGCTCTGGAAAAGAAAATGGAAAAGACA  CAAGTTACCTCATCCTCACCTTCA  GATAATAGAACCAGAGCCATCGTATG  CTGCTCAAACAAATCCAAGTGTTC  CCATTTCCGTTGTTTCCTTTAGTAG  AATTACTCAAGTGTCGCTCCTATTTCT  ACCAATAATAGCAGCACCGAAAG  GCACGTCACCTCTACGTTATGG  CGATGACCGGGCACTTG  TTGCTTATGATAATGCTCCATACGA  TACCCCACAATCTTGGCAAGT  ATAGCGAACTTGTTACTAGT  GAACCAGAGCCATCGTATGT  AACCATAGCAACAACCGGTT  GAACCAGATCCGTCAAATGT  CACTGAACCTTCCCCAGTTG  GGTTGCATGAGTGGAACTGA  TTGCTTATGATAATGCTCCATACGA  TTGAGACCACCAACCAAACA |

^a^The restriction sites are underlined.


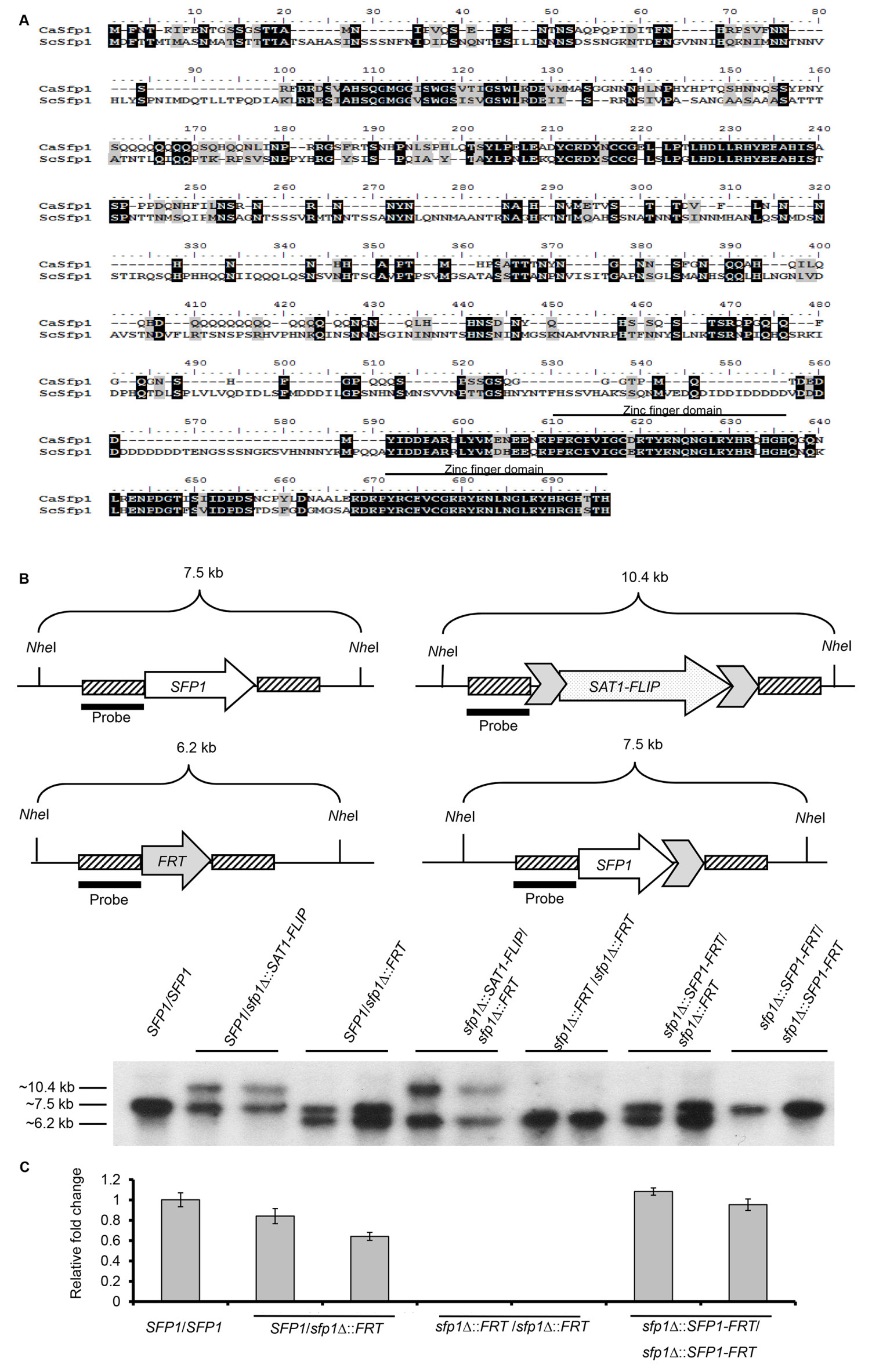


**Supplemental Figure A. Construction of the *sfp1*∆/*sfp1*△ and *SFP1*-reintegrated strains.** (A) The amino acid sequence of *C*. *albicans* Sfp1 was aligned with *S. cerevisiae* Sfp1 using Clustal W2. We observed 31% identity and 40% similarity between the two proteins. Moreover, both Sfp1 genes contain zinc finger DNA binding domains in their C-terminal regions. (B) Southern blot analysis of the WT, *sfp1*∆**/***sfp1*△ and *SFP1*-reintegrated strains. The genomic DNA was digested with *Nhe*I. The specific probe was generated via PCR using the primer pair CaSFP1uF-KpnI and CaSFP1uR-XhoI. (C) The expression of *SFP1* in the WT, *SFP1*/*sfp1*∆, *sfp1*∆/*sfp1*∆ and *sfp1*∆::*SFP1*/*sfp1*∆::*SFP1* strains was detected via real-time qPCR.


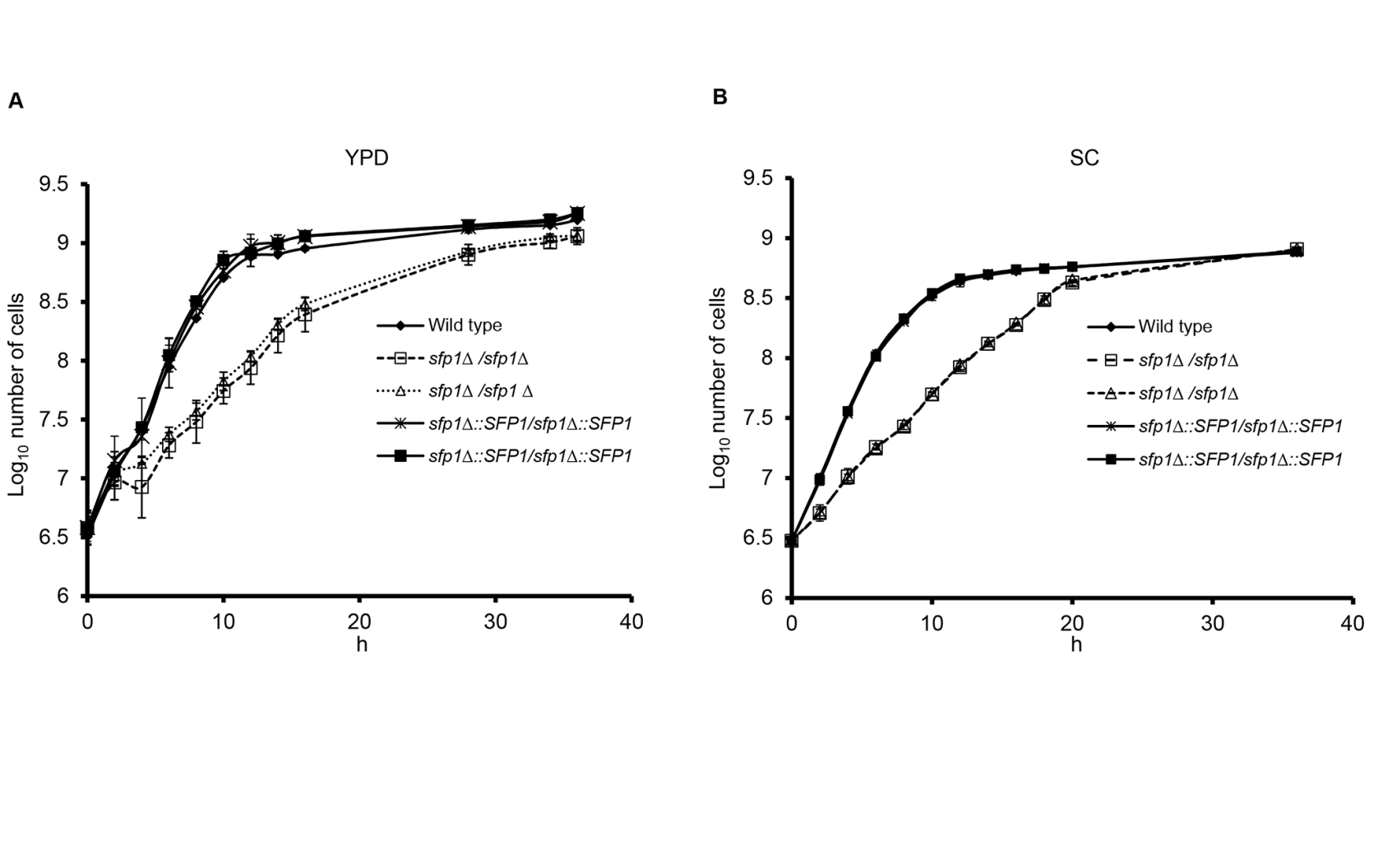


**Supplemental Figure B. *C. albicans* *SFP1*-deletion strains exhibit a growth delay compared with the wild type and *SFP1*-reintegrated strains.** Cells of the WT, *sfp1*∆/*sfp1*∆ and *SFP1*-reintegrated strains were incubated overnight in YPD medium, subcultured in freshly prepared (A) YPD or (B) SC medium (initial cell density OD_600nm_ ~ 0.1) and incubated at 30ºC. Samples were collected at different times. The presented results are the average of two independent experiments and are presented as the Log number of cells per ml.


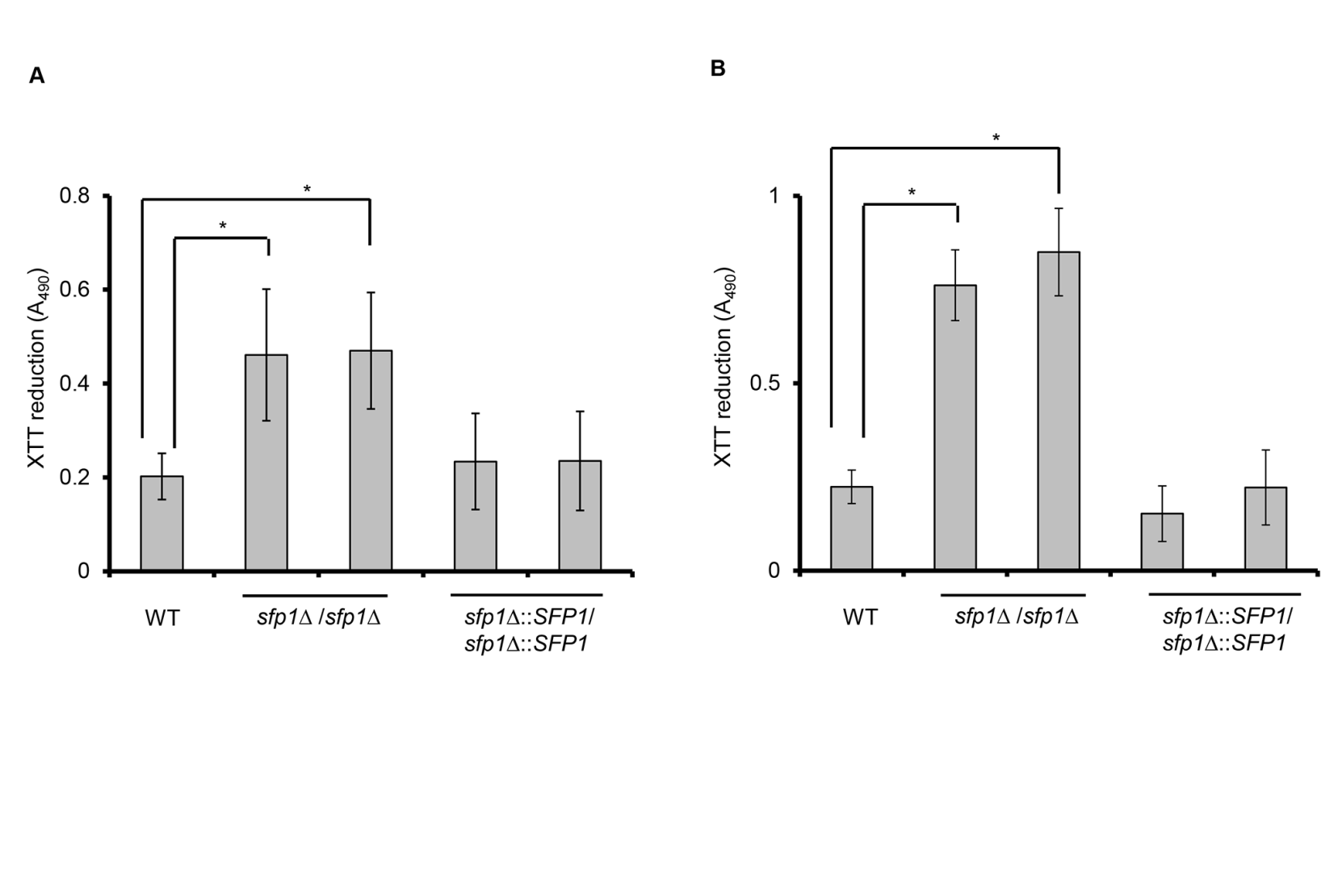


**Supplemental Figure C. Deletion of *SFP1* enhances *C. albicans* adhesion and biofilm formation in YPD medium.** (A) Cell adhesion was assessed in a 24-well polystyrene microplate in YPD medium at 37°C with 5% CO_2_ for 1 h. Cell adhesion was determined using the XTT reduction assay. The results are presented as the mean ± SD from three independent experiments. *p < 0.05 for *sfp1*△/*sfp1*△ vs. WT cells. (B) Biofilm formation was assessed using the XTT reduction assay. Biofilm formation was performed in a 24-well polystyrene microplate in YPD medium at 37°C with 5% CO_2_ for 24 h. The results are presented as the mean ± SD from three independent experiments. *p < 0.05 for *sfp1*△/*sfp1*△ vs. WT cells.


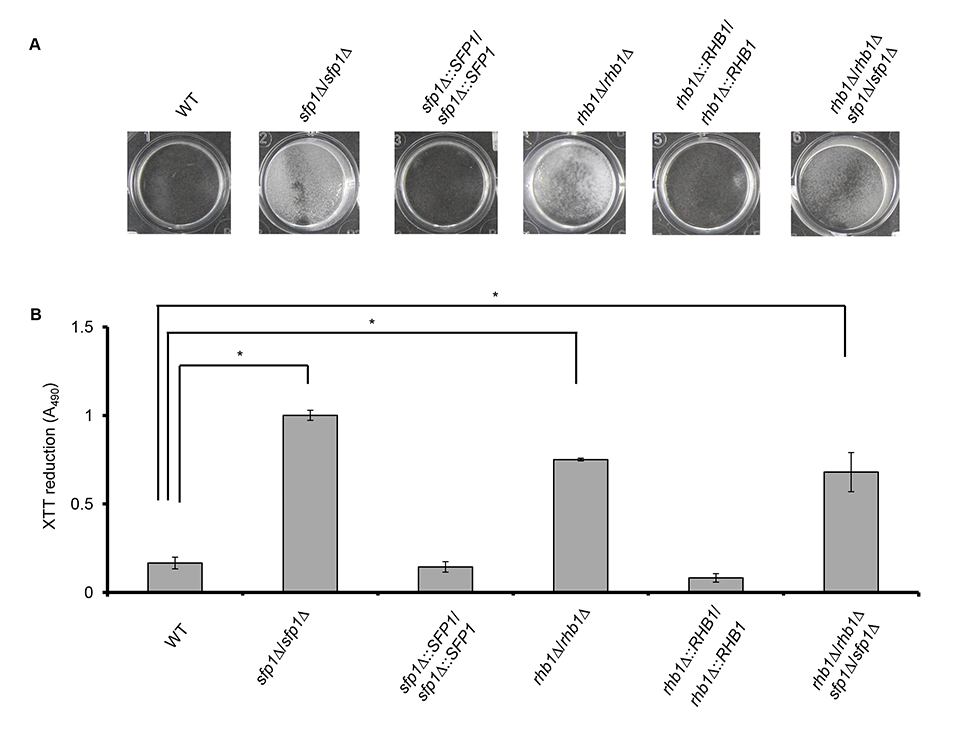


**Supplemental Figure D. Deletion of *RHB1* enhances *C. albicans* biofilm formation.** (A) Biofilms were formed on the surface of a 24-well polystyrene microplate in SC medium at 37°C with 5% CO_2_ for 24 h. (B) Biofilm formation was assessed using the XTT reduction assay. The results are presented as the mean ± SD from three independent experiments. *p < 0.05 for mutants vs. WT cells.


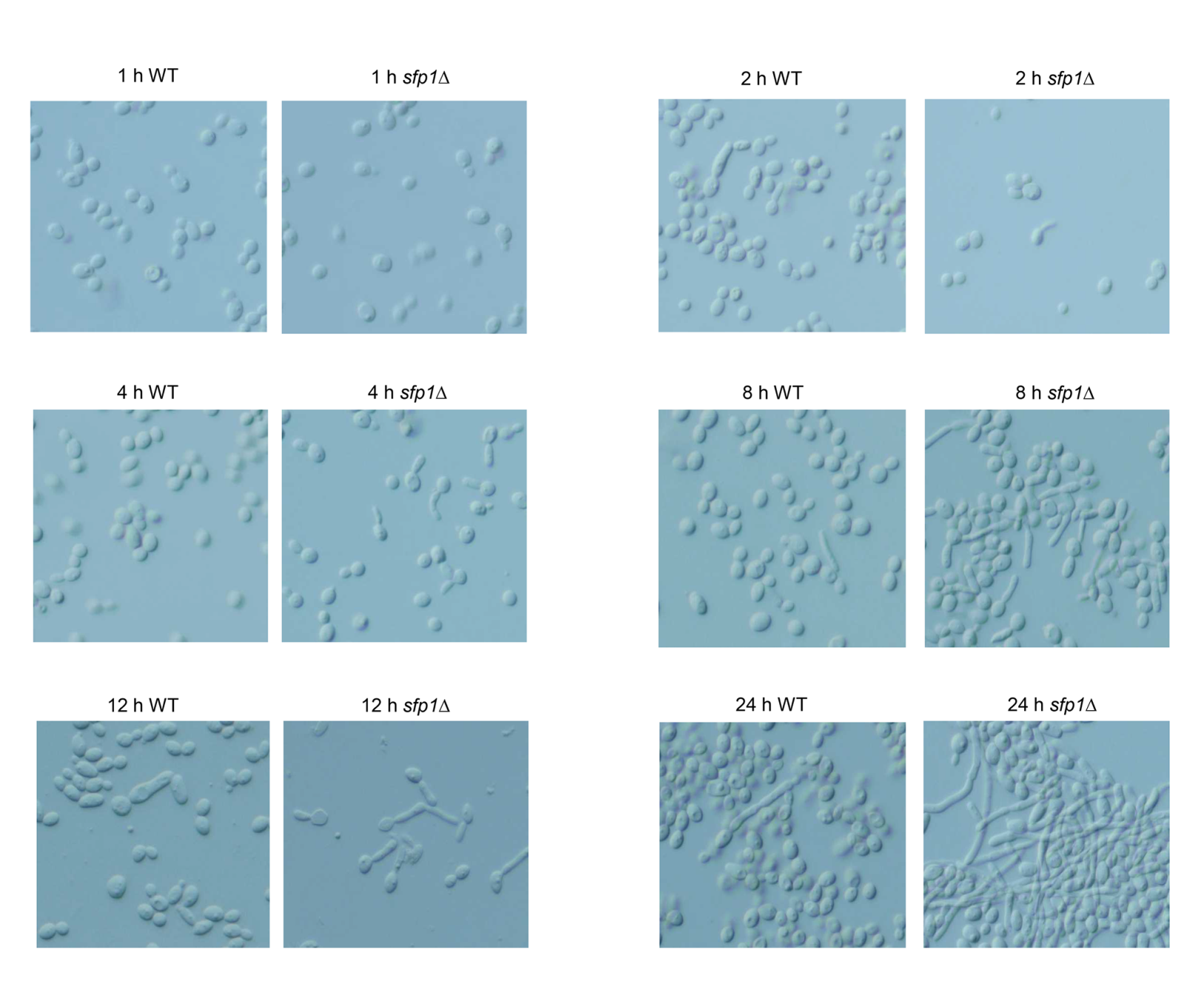


**Supplemental Figure E. Cell morphology during the process of biofilm formation in the WT and *sfp1*∆/*sfp1*△ strains.** The cells were incubated in SC medium at 37°C with 5% CO_2_. At different time intervals, the adherent cells were washed and cell morphology was visualized at a 400× magnification.


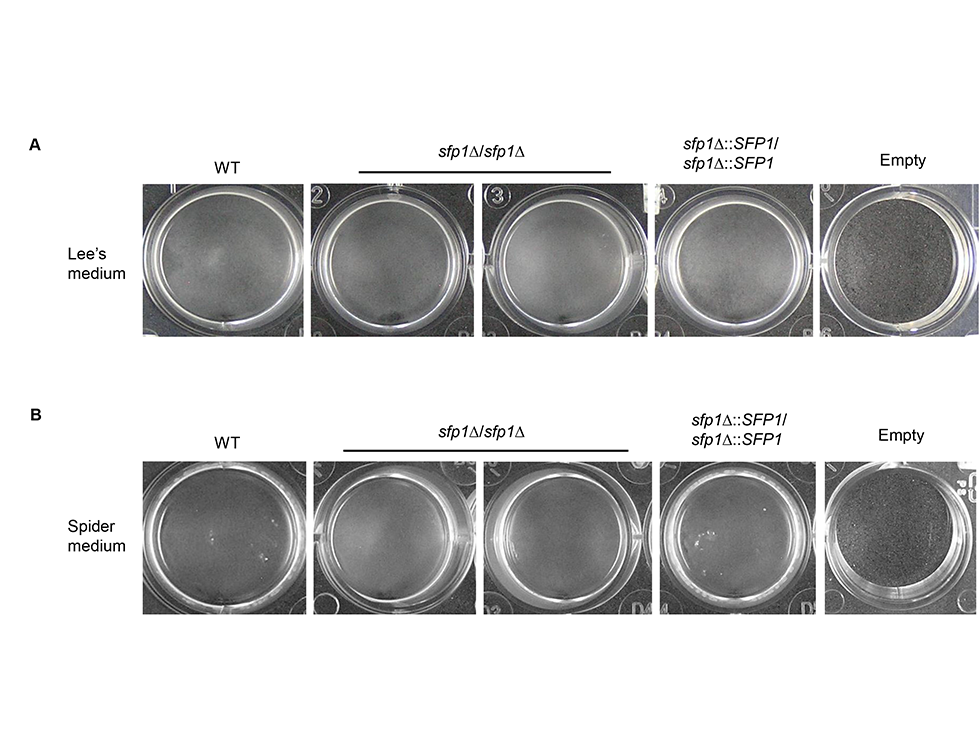


**Supplemental Figure F. WT, *sfp1*Δ/*sfp1*Δ and *SFP1*-reintegrated strains all could form biofilm in Lee’s and Spider media.** Biofilms were formed on the surface of a 24-well polystyrene microplate in (A) Lee’s and (B) Spider media at 37°C with 5% CO_2_ for 24 h.


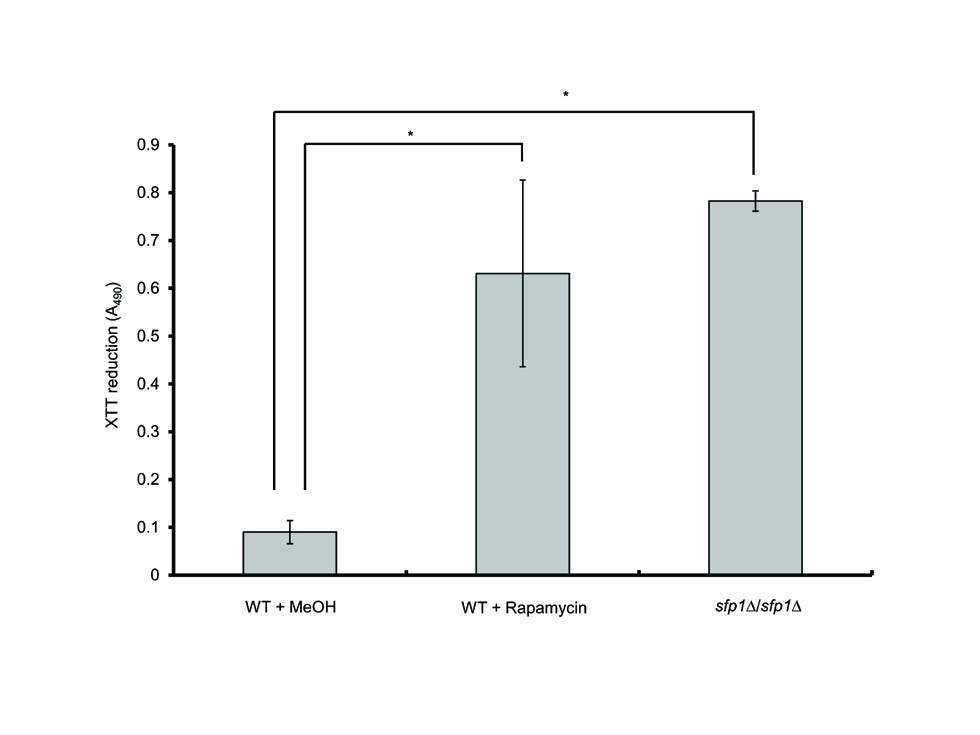


**Supplemental Figure G. Inhibition of Tor1 enhances *C. albicans* biofilm formation.** WT cells were treated with 75 ng/ml of rapamycin (prepared with methanol (MeOH)) or an equal volume of MeOH, which was the vehicle control. Biofilms were formed in 24-well plates in SC medium at 37°C with 5% CO_2_ for 24 h. Biofilm formation was determined using the XTT reduction assay. The results are presented as the mean ± SD from three independent experiments. *p < 0.05 for WT (with rapamycin treatment) vs. WT (without rapamycin treatment) and *sfp1*△/*sfp1*△ vs. WT.
